# A novel tRNA-derived fragment AS-tDR-007333 promotes the malignancy of NSCLC via the HSPB1/MED29 and ELK4/MED29 axes

**DOI:** 10.1186/s13045-022-01270-y

**Published:** 2022-05-07

**Authors:** Wenhan Yang, Kaiping Gao, Youhui Qian, Yongyi Huang, Qin Xiang, Cheng Chen, Qianqian Chen, Yiling Wang, Fuyuan Fang, Qihan He, Siqi Chen, Juan Xiong, Yangchao Chen, Ni Xie, Duo Zheng, Rihong Zhai

**Affiliations:** 1 School of Public Health, Shenzhen University Health Science Center, 1066 Xueyuan Ave., Shenzhen, 518055 China; 2 Department of Thoracic Surgery, The First Affiliated Hospital of Shenzhen University, 3002 West Shungang Road, Shenzhen, 518035 China; 3 Guangdong Provincial Key Laboratory of Genome Stability and Disease Prevention, International Cancer Center, Shenzhen University Health Science Center, Shenzhen, China; 4 Faculty of Medicine, The Chinese University of Hong Kong, Rm508A, Lo Kwee-Seong Integrated Biomedical Sciences Bldg, Shatin, NT, Hong Kong, China; 5 Department of Thoracic Surgery, Shenzhen University General Hospital, 1098 Xueyuan Ave., Shenzhen, 518055 China

**Keywords:** tRNA-derived fragments, AS-tDR-007333, MED29, HSPB1, ELK4, Non-small cell lung cancer

## Abstract

**Background:**

Transfer RNA-derived fragments (tRFs) are a new class of small non-coding RNAs. Recent studies suggest that tRFs participate in some pathological processes. However, the biological functions and mechanisms of tRFs in non-small cell lung cancer (NSCLC) are largely unknown.

**Methods:**

Differentially expressed tRFs were identified by tRF and tiRNA sequencing using 9 pairs of pre- and post-operation plasma from patients with NSCLC. Quantitative real-time PCR (qRT-PCR) and fluorescence in situ hybridization (FISH) were used to determine the levels of tRF in tissues, plasma, and cells. Gain- and loss-of-function experiments were implemented to investigate the oncogenic effects of tRF on NSCLC cells in vitro and in vivo. Chromatin immunoprecipitation (ChIP), luciferase reporter, RNA pulldown, mass spectrum, RNA immunoprecipitation (RIP), Western blot, co-immunoprecipitation (Co-IP) assays, and rescue experiments were performed to explore the regulatory mechanisms of tRF in NSCLC.

**Results:**

AS-tDR-007333 was an uncharacterized tRF and significantly up-regulated in NSCLC tissues, plasma, and cells. Clinically, AS-tDR-007333 overexpression could distinguish NSCLC patients from healthy controls and associated with poorer prognosis of NSCLC patients. Functionally, overexpression of AS-tDR-007333 enhanced proliferation and migration of NSCLC cells, whereas knockdown of AS-tDR-007333 resulted in opposite effects. Mechanistically, AS-tDR-007333 promoted the malignancy of NSCLC cells by activating MED29 through two distinct mechanisms. First, AS-tDR-007333 bound to and interacted with HSPB1, which activated MED29 expression by enhancing H3K4me1 and H3K27ac in MED29 promoter. Second, AS-tDR-007333 stimulated the expression of transcription factor ELK4, which bound to MED29 promoter and increased its transcription. Therapeutically, inhibition of AS-tDR-007333 suppressed NSCLC cell growth in vivo.

**Conclusions:**

Our study identifies a new oncogenic tRF and uncovers a novel mechanism that AS-tDR-007333 promotes NSCLC malignancy through the HSPB1-MED29 and ELK4-MED29 axes. AS-tDR-007333 is a potential diagnostic or prognostic marker and therapeutic target for NSCLC.

**Supplementary Information:**

The online version contains supplementary material available at 10.1186/s13045-022-01270-y.

## Background

Lung cancer is the most common cancer type, and non-small cell lung cancer (NSCLC) accounts for up to 85% of all lung cancer cases [1]. Despite considerable progressions in diagnostic and therapeutic approaches in recent years, the overall 5-year survival rate for patients with NSCLC is still unsatisfactory [2]. Although extensive studies have shown that multiple oncogenes and tumor suppressor genes are involved in the pathogenesis of NSCLC, the molecular basis of NSCLC carcinogenesis remains incompletely elucidated. Therefore, it is still necessary to explore unknown molecular mechanisms in NSCLC to identify new diagnostic and therapeutic targets.

tRNA-derived fragments (tRFs) are a novel class of small non-coding RNAs (sncRNAs) produced by the specific cleavage of mature or precursor transfer RNAs (tRNAs) [3]. Based on its length, cleavage position, and sequence matched on tRNAs, tRFs are broadly classified into five subtypes: tRF-5, tRF-3, tRF-2, tRF-1, and tRNA halves (tiRNA) [4]. tRF-5 is produced by cleavage of the 5′ end in the D-loop; tRF-3 is derived from the 3′ end in the TψC-loop and contains the CCA modification; the tRF-2 comprises only the anticodon stem and loop tRNA; tRF-1 is generated from the beginning of the 3′ end of precursor tRNA and is characterized by carrying poly-U residues at the 3′ terminus; and the tiRNA is generated by specific cleavage in the anticodon loops of the mature tRNA [5]. tRFs were originally viewed as non-functional degradation products of tRNA found in next generation sequencing (RNA-seq) datasets. However, the recurrence of reads matching specific domains of mature tRNAs suggested that these sncRNAs could be functional [6]. Indeed, there is emerging evidence that tRFs participate in various molecular processes such as gene silencing, RNA processing, protein biosynthesis and oncogenic transformation [7, 8]. Several tRFs have also been associated with proliferation, migration, and invasion in some types of cancer cells [9–12]. However, whether and how tRFs could engage in NSCLC tumorigenesis remain largely unknown.

In this study, we identified a novel tRF termed AS-tDR-007333. We found that AS-tDR-007333 was up-regulated in NSCLC and its up-regulation was associated with the development and progression of NSCLC. We revealed that AS-tDR-007333 promoted NSCLC tumorigenesis via a dual HSPB1- and ELK4-MED29 regulatory mechanisms. Our findings indicated that tRF-activated regulatory processes may represent an additional molecular mechanism in NSCLC and AS-tDR-007333 may serve as a potential therapeutic target for NSCLC.

## Methods

### Clinical samples and cell lines

This study was approved by the Medical Ethics Committee of Shenzhen University Health Science Center (Approved no. 2016002). Written informed consents were obtained from all participants. NSCLC subjects were recruited from patients who underwent surgical resection in the Department of Thoracic Surgery at the First Affiliated Hospital of Shenzhen University, China. NSCLC was diagnosed according to the criteria of Lung Cancer Stage Classification (The Eighth Edition). No patients received any kind of therapy or had history of other malignancies prior to surgery. Controls were healthy subjects who took routine medical examination at the same hospital and were clinically confirmed without cancer or carrying a previous diagnosis of any cancer. The human NSCLC cell lines (PC9, A549, HCC827, and NCI-H226) and the normal human bronchial epithelial cells (BEAS-2B) were obtained from the Cell Bank of Chinese Academy of Biological Sciences (Shanghai, China). All cell lines were authenticated through short tandem repeat (STR) DNA profiling. No contamination of mycoplasma was found in these cell lines.

### tRFs and tiRNA sequencing

Total RNA was extracted from plasma using the TRIzol® reagent (Invitrogen, MA, USA). To remove RNA modifications that may interfere with construction of small RNA library, total RNA samples were firstly pretreated with the following reagents: 3′-aminoacyl (charged) deacylation to 3′-OH for 3′ adaptor ligation, 3′-cP (2′,3′-cyclic phosphate) removal to 3′-OH for 3′ adaptor ligation, 5′-OH (hydroxyl group) phosphorylation to 5′-P for 5′-adaptor ligation, m1A and m3C demethylation for efficient reverse transcription. The pretreated total RNA samples were then subjected to library preparation using the NEBNext® Multiplex Small RNA Library Prep Set for Illumina® kit (New England BioLabs, MA, USA). Briefly, RNA was ligated with 3′ and 5′-adapters, and cDNA was synthesized, followed by PCR amplification. The completed libraries were sequenced on Illumina NextSeq 500 system (Illumina, CA, USA) at Aksomics Inc. (Shanghai, China), using the NextSeq 500/550 V2 kit (#FC-404-2005, Illumina).

The sequencing quality was examined by FastQC software, and trimmed reads (pass Illumina quality filter, trimmed 3′-adaptor bases by cut adapt) were aligned to mature-tRNA and pre-tRNA sequences in the tRNA database (http://GtRNAdb.ucsc.edu) using the Novo Align software (v2.07.11). The unmapped reads were aligned to other corresponding databases (mRNA/rRNA/snRNA/snoRNA/piRNA/miRNA). The tRF and tiRNA expression levels were measured and normalized to the number of transcripts per million of total aligned tRNA reads (TPM). Paired *P* value < 0.05 was considered statistically significant.

### Animal experiment

Animal study was approved by the Animal Ethical and Welfare Committee of Shenzhen University Health Science Center (Approved No. AEWC-2020007). Agomir-AS-tDR-007333-inhibitor and its NC were synthesized and labeled with cy5 by Ribobio Co. (Guangzhou, China). Four-week-old BALB/C nude mice were injected subcutaneously with 3 × 10^6^ A549 cells [13], and the established xenografts were monitored by IVIS Lumina K II in vivo imaging system (PerkinElmer). Two weeks after injection, mice were randomly divided into three groups: (A) Inhibitor, (B) Inhibitor-NC, (C) blank control (PBS), with 6 mice in each group. 5 nmol Inhibitor, NC, or equal volume of PBS were locally injected into the tumor mass once every 3 days, respectively. The tumor sizes were measured twice a week and were calculated using the equation V = length × width^2^/2 (V, volume). Two weeks after inhibitor, NC, or PBS injection, the mice were killed and the xenograft tumors were excised, formalin-fixed and paraffin-embedded, or stored at − 80 °C for further analyses.

### Tissue microarray (TMA) and fluorescence in situ hybridization (FISH)

TMA was produced from paraffin-embedded samples by Outdo Biotech (HLugA180Su04, Shanghai, China). The probe and the FISH Kit were ordered from Boster Biological Technology (Wuhan, China). Briefly, the TMA was dewaxed in xylene and rehydrated through gradient alcohol, digested using pepsin, hybridized with the probe, incubated with anti-Digoxin-AP (Roche, Basel, Switzerland) and then stained with 3,3′-diaminobenzidine (DAB). Images were taken using a fluorescence microscope (Nikon Corporation, Tokyo, Japan). The expression of AS-tDR-007333 was quantified using a visual grading system based on the degree of staining (Additional file 2: Fig. S12). The intensity of staining was divided into four grades: 0, negative; 1, weak; 2, moderate; and 3, strong. The positive cell percentages were classified as: 0, negative; 1, 1–25%; 2, 26–50%; 3, 51–75%; and 4, > 75%. A weighted staining score was calculated by multiplying the positive cells percentage and the grade of the staining intensity. Finally, all samples were assigned to two levels according to the score: < 3, low expression; ≧3, high expression.

### RNA pulldown and mass spectrometry

Biotin-labeled AS-tDR-007333 probe and control probe were transcribed in vitro using the transcript Aid T7 High Yield Transcription Kit (Thermo Scientific, Shanghai, China) according to the manufacture’s guidelines. PC9 cells were cultured in RIPA Lysis and Extraction Buffer (Thermo Scientific), and the supernatant was incubated with biotinylated probes and then mixed with Dynabeads MyOne Streptavidin C1 beads (Thermo Scientific, Shanghai, China). Then, the RNA–protein mixture was boiled in SDS buffer, subjected to SDS-PAGE and silver staining. Protein bands with significant differences were cut and subjected to protein mass spectrometry on the Q Exactive Mass spectrometry System (Thermo Scientific). Raw mass spectrometry data were processed using MM File Conversion, and protein identifications were analyzed using Mascot v2.6.0 against the Human UniProt database.

### RNA-seq

RNA-sequencing was carried out to identify the target genes regulated by AS-tDR-007333. PC9 cells were transfected with AS-tDR-007333 or NC for 48 h. Thereafter, RNA was isolated from the treated PC9 cells using the TRIzol reagents (Invitrogen, Shanghai, China). RNA-seq libraries were prepared using the Illumina KAPA Stranded RNA-Seq Library Prep Kit (Illumina, CA, USA). Sequencing was performed on an Illumina HiSeq 4000 by KangChen Biotech (Shanghai, China). Sequencing reads were trimmed using StringTie and mapped to human genome database (GRCh37) by the Hisat2 software. Differential expression and normalized read counts (FPKM, Fragments per kilobase of gene/transcript model per million mapped fragments) were calculated using the Ballgown software.

### Chromatin immunoprecipitation (ChIP)

The ChIP assay was performed using the ChIP Kit (Beyotime, Shanghai, China). Briefly, cells were treated with 1% formaldehyde solution for 10 min and quenched in glycine for 5 min at room temperature to generate DNA–protein cross-links. Cell lysates were then sonicated to produce chromatin fragments of 200–1000 bp and immunoprecipitated with anti-HSPB1, anti-ELK4, or IgG antibodies. Immunoprecipitated DNAs were analyzed by qRT-PCR. Information for primers and antibodies is listed in Additional file 1: Table S2.

### Dual-luciferase reporter assay

The full-length promoter of MED29 carrying mutant or wild-type sequences was cloned into pGLO4.10 vectors (Promega, MWI, USA), respectively, and co-transfected with ELK4 overexpression vector or mock vector into PC9 cells, using Lipofectamine TM 2000 (Invitrogen, CA, USA). After 48 h of culture, the activities of firefly and Renilla luciferase were measured using the dual-luciferase reporter gene assay system (Beyotime, Shanghai, China) in accordance with the manufacturer’s protocols.

### Additional information on methods

See Supplementary materials for additional information on methods.

## Results

### Circulating tRFs are differentially expressed between pre- and post-operation plasma samples in patients with NSCLC

**Fig. 1:**
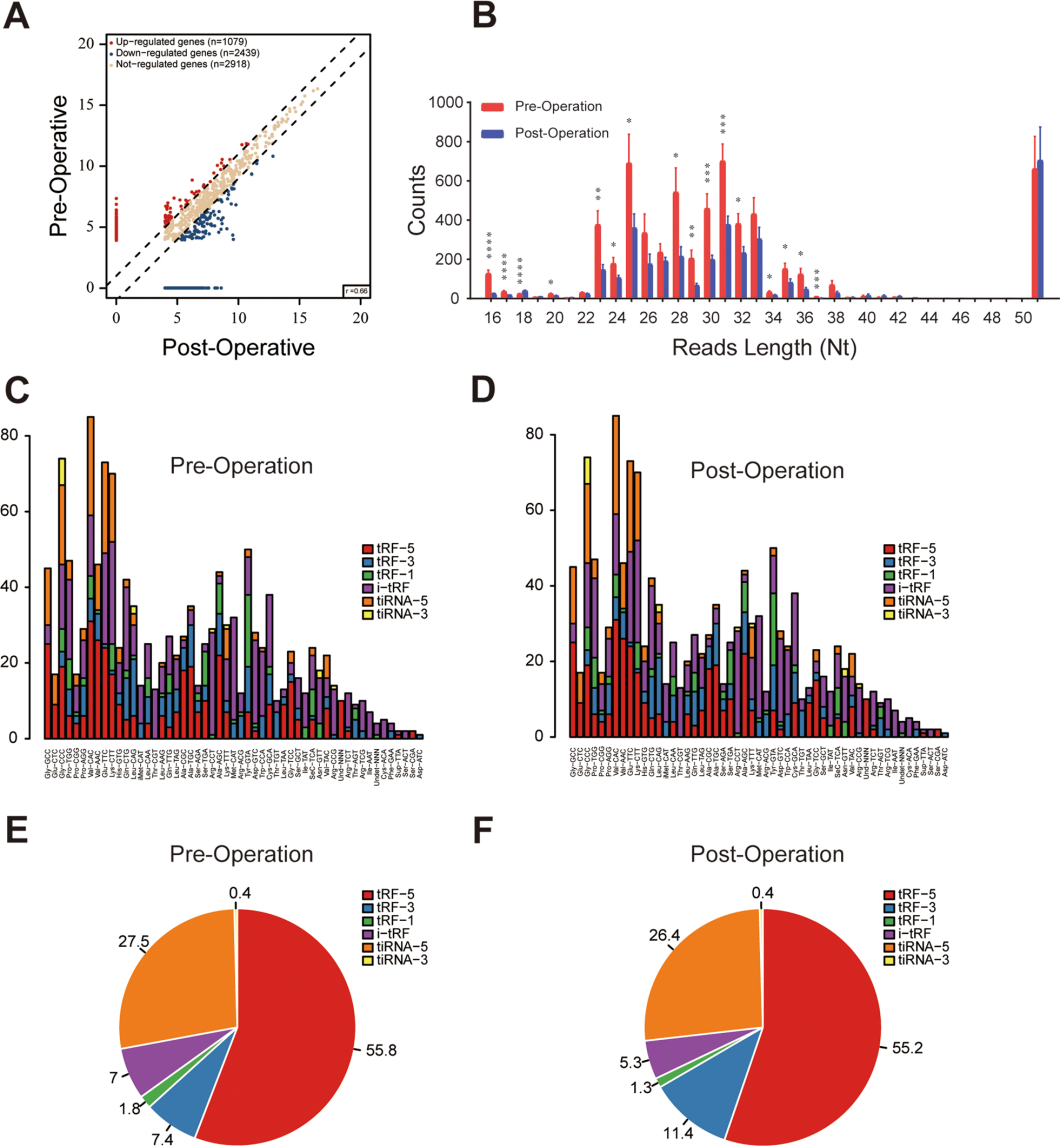
Characteristics of plasma tRF profiles in patients with NSCLC. **A** Scatter plot analysis revealed the differentially expressed tRFs between pre- and post-operation plasma samples. The red and blue points denote the significantly up- or down-regulated tRFs between two groups. **B** Length-wise distribution of tRFs between pre- and post-operation plasma samples in patients with NSCLC, showing the reads of most length-based tRF types in plasma were significantly decreased after removal of tumors. **C**, **D** Stacked plot for the number of tRFs and tiRNAs derived from the same tRNA: The X- and Y-axis represented the tRNAs and the number of different kinds of tRFs and tiRNAs derived from the same tRNA, respectively. **E**, **F** The pie charts indicated that tRF-5 is the major type of plasma tRFs in patients with NSCLC. *P < 0.05; **P < 0.01; ***P < 0.001; ****P < 0.0001

To identify and characterize differentially expressed circulating tRFs in NSCLC, we compared the tRF expression profiles between nine pairs of pre- and post-operation plasma samples from patients with NSCLC. tRF and tiRNA sequencing revealed that tRF expression profiles differed substantially between pre- and post-operation plasma samples (Fig. 1A). The lengths of circulating tRFs ranged from 15 to 50 nucleotides (nt), with 53.97% and 30.11% of the tRFs between 20 and 23 (nt), and 31 to 33 nt, respectively (Fig. 1B). Notably, the expression levels of most tRFs in post-operation plasma were significantly lower than that in pre-operation plasma samples (Fig. 1B), suggesting that up-regulated plasma tRFs were associated with the existence of tumors in NSCLC. The stacked plot indicated that one type of tRF or tiRNA can be produced from different tRNAs by cleavage into the fragments with identical sequences (Fig. 1C, D). Pie plot analysis revealed that most plasma tRFs were derived from the 5′ end of tRNAs; similarly, tiRNA series belonged more to tiRNA-5 (Fig. 1E, F).

### tRF AS-tDR-007333 is up-regulated in NSCLC

**Fig. 2:**
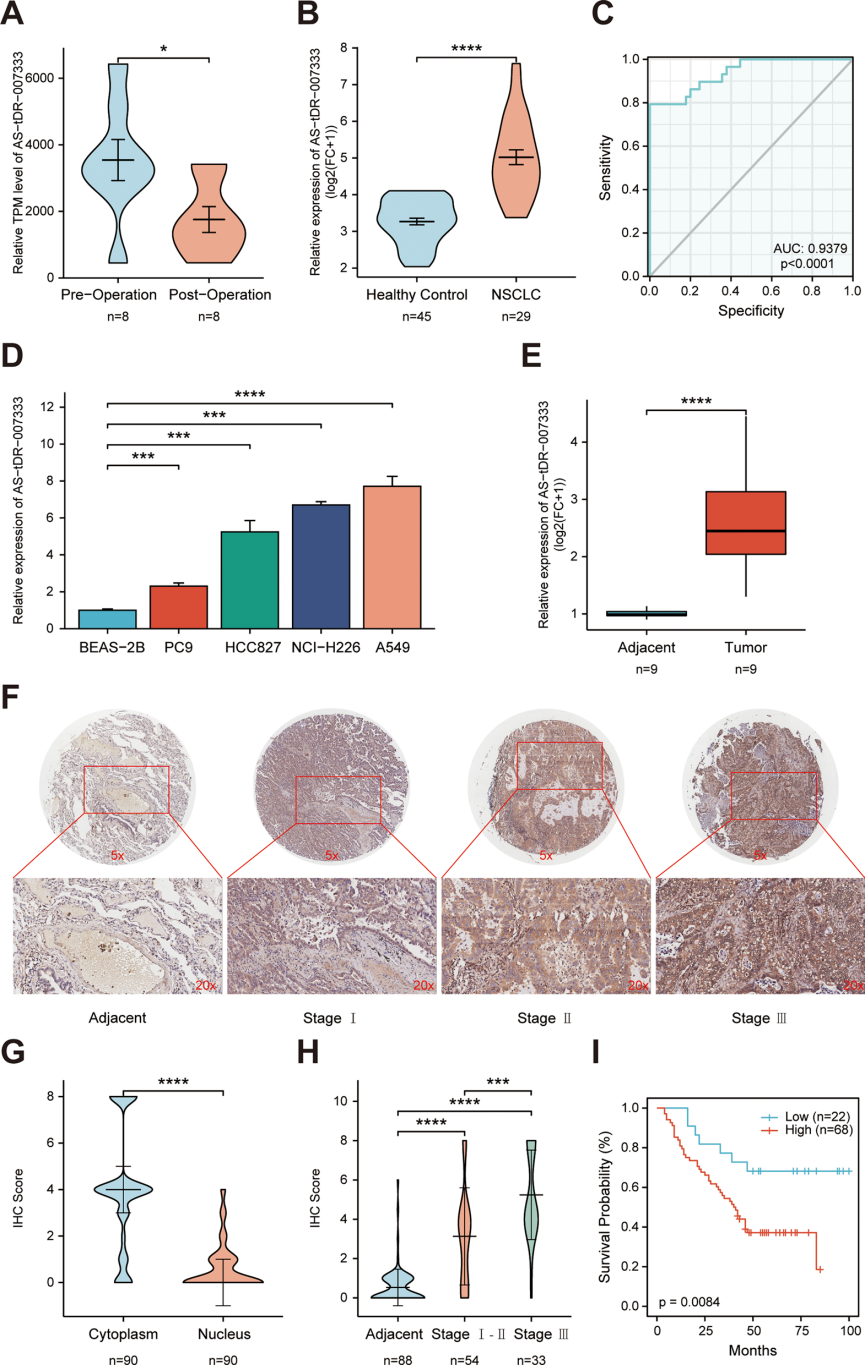
tRF AS-tDR-007333 is up-regulated in NSCLC and associated with worse prognosis in patients with NSCLC. **A** AS-tDR-007333 expression level in pre-operation plasma was higher than that in post-operation plasma samples. **B** Expression levels of plasma AS-tDR-007333 in patients with NSCLC (n = 29) were significantly (*P* < 0.001) higher than that in healthy controls (n = 45). **C** The ROC curve analysis on plasma AS-tDR-007333 level for discriminating NSCLC patients from healthy controls (AUC = 0.9379, sensitivity = 97.78%, specificity = 79.31%). **D** The expression levels of AS-tDR-007333 in NSCLC cells were higher than that in normal bronchial epithelial cells (BEAS-2B). **E** qPCR assay revealed that AS-tDR-007333 expression was increased significantly in NSCLC tumor tissues, compared with the corresponding adjacent tissues. **F**, **H** FISH assay demonstrated that AS-tDR-007333 expression level was higher in NSCLC tumor tissues (n = 90) than that in the adjacent tissues (n = 86) and increased as NSCLC progressed to more advanced stages. The purple staining represented positive signal. **G** FISH analysis showed that AS-tDR-007333 was mainly enriched in the cytoplasm. **I** Higher AS-tDR-007333 levels were associated with shorter overall survival in NSCLC patients (log-rank *P* value = 0.008; cutoff value: score = 3.0). *P < 0.05; **P < 0.01; ***P < 0.001; ****P < 0.0001

With a cutoff criteria of fold change ≧2.0 and *P* < 0.05, we identified 4 up-regulated tRFs and 4 down-regulated tRFs in pre-operation plasma samples compared to that in post-operation plasma samples. Among them AS-tDR-007333 was a novel tRF that has not been previously reported in tRF databases. The AS-tDR-007333 was 28 nt long and cleaved at site 1 to 28 on the 5′ end of tRNA-Gly-GCC (Additional file 2: Fig. S1a-c). Using qRT-PCR assay, it was confirmed that the expression levels of AS-tDR-007333 in pre-operation plasma were significantly (*P* = 0.0312) higher than that in post-operation plasma samples in patients with NSCLC (Fig. 2A). qRT-PCR analysis in additional sample set consisting of plasma from NSCLC patients (n = 29) and healthy controls (n = 45) showed that plasma AS-tDR-007333 concentration in NSCLC patients was significantly higher than that in healthy controls (Fig. 2B). Receiver operating characteristic (ROC) analysis indicated that the area under the curve (AUC) was 0.9379 (sensitivity = 97.78%, specificity = 79.31%) (Fig. 2C), suggesting that plasma AS-tDR-007333 level had high potential to serve as diagnostic biomarker for NSCLC. In addition, AS-tDR-007333 expression levels in NSCLC cell lines (PC9, HCC827, NCI-H226, and A549) were also higher than that in normal human bronchial epithelial cells (BEAS-2B) (Fig. 2D). Moreover, the expression levels of AS-tDR-007333 in NSCLC tumor tissues (n = 9) were significantly (*P* < 0.0001) higher than that in the adjacent normal tissues (n = 9) (Fig. 2E). Taken together, multiple lines of evidence clearly indicated that AS-tDR-007333 was up-regulated in NSCLC and might play a critical role in the pathogenesis of NSCLC.

### Higher expression of AS-tDR-007333 is associated with poor prognosis of NSCLC

To assess the clinical significance of AS-tDR-007333 in NSCLC patients, we used FISH to determine AS-tDR-007333 expression levels in NSCLC tumor tissues and adjacent tissues. The results showed that AS-tDR-007333 level was positively correlated with TNM stages (Fig. 2F, H), suggesting that AS-tDR-007333 levels were associated with the progression of NSCLC. Moreover, AS-tDR-007333 was more abundant in the cytoplasm than in the nucleus (Fig. 2G, Additional file 1: Tables S3, S4). Thus, cytoplasm staining scores of AS-tDR-007333 were used to assess the relationships between the levels of AS-tDR-007333 and the outcomes of NSCLC. When patients were divided into two groups based on the cytoplasm score of AS-tDR-007333 (score≧3, high expression; score < 3, low expression), Kaplan–Meier survival analysis revealed that higher AS-tDR-007333 levels were associated with lower overall survival (OS) in NSCLC patients (log-rank test, *P* = 0.008, Fig. 2I). Furthermore, multivariate Cox regression analysis confirmed that higher expression of AS-tDR-007333 was significantly associated with shorter survival time in NSCLC (HR = 2.288; 95%CI, 1.0203–5.1310; *P* = 0.04) (Additional file 1: Table S5). Thus, clinical data strongly suggested that higher AS-tDR-007333 level was associated with poor prognosis in NSCLC patients.

### AS-tDR-007333 promotes proliferation and migration of NSCLC cells

**Fig. 3:**
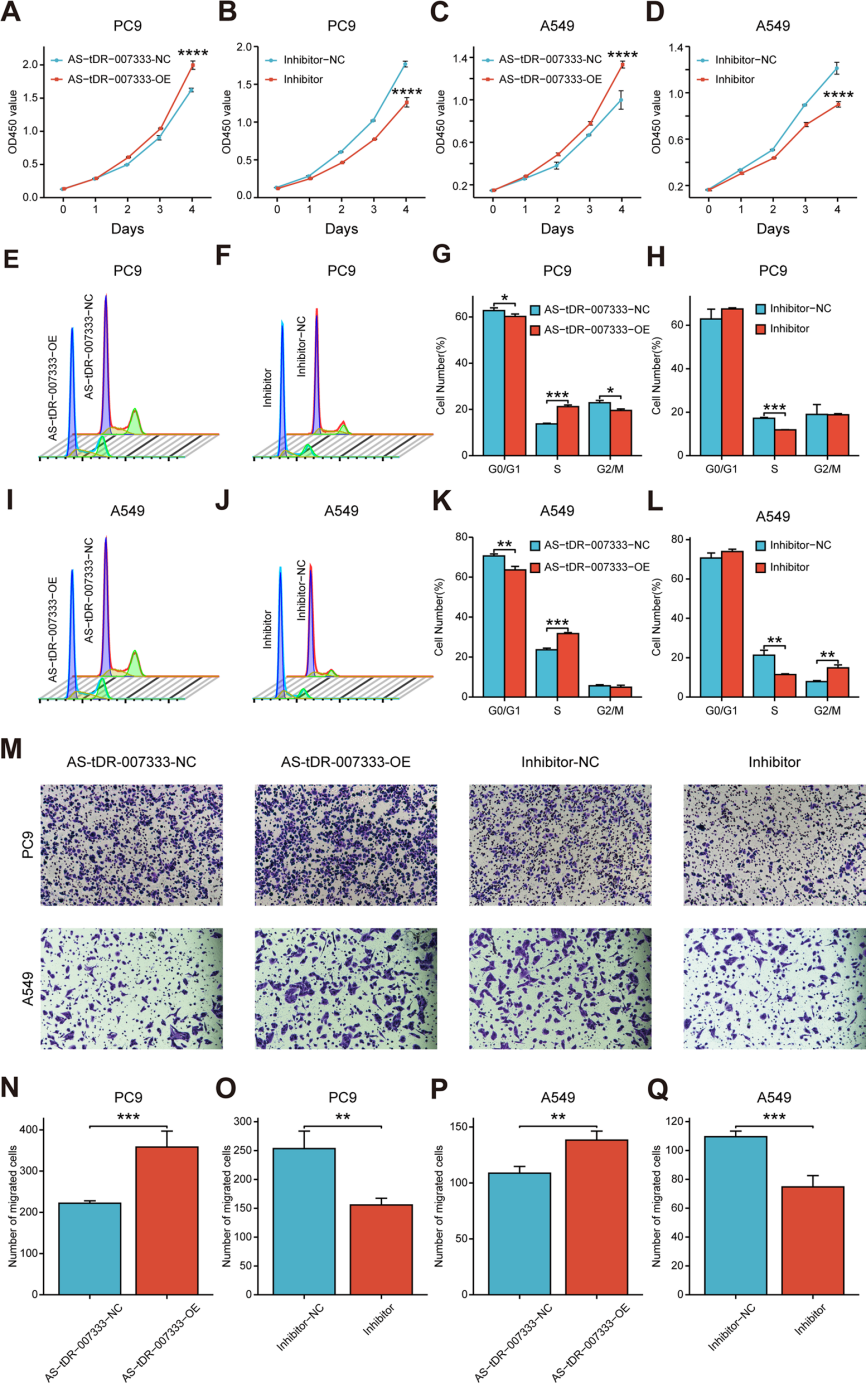
AS-tDR-007333 promotes proliferation and migration capacity of NSCLC cells. **A**, **C** Overexpression of AS-tDR-007333 promoted proliferation rates of A549 and PC9 cells. **B**, **D** Knockdown of AS-tDR-007333 with inhibitor suppressed cell proliferation in A549 and PC9 cells. **E**, **G** Cell cycle analysis revealed that the percentages of S phase cells in AS-tDR-007333-overexpressed PC9 cells were increased as compared with their negative controls. **F**, **H** Knockdown of AS-tDR-007333 reduced the rate of S phase cells in PC9 cell line. **I**, **K** Up-regulation of AS-tDR-007333 increased the number of S phase cells in A549 cells. **J**, **L** Knockdown of AS-tDR-007333 reduced the rate of S phase cells in A549 cells. **M**–**Q** Transwell assays showed that up-regulation of AS-tDR-007333 increased the numbers of migrated cells, but knockdown of AS-tDR-007333 reduced the numbers of migrated cells. *P < 0.05; **P < 0.01; ***P < 0.001; ****P < 0.0001

To evaluate the biological functions of AS-tDR-007333 in NSCLC, we conducted gain- and loss-of-function experiments in NSCLC cells. The CCK-8 assay showed that overexpression of AS-tDR-007333 significantly promoted cell proliferation, whereas knockdown of AS-tDR-007333 significantly suppressed NSCLC cell proliferation (Fig. 3A–D). To examine whether the effect of AS-tDR-007333 on the proliferation of NSCLC cells reflected cell cycle transition, flow cytometric analysis was carried out to investigate cell cycle progression. The results showed that PC9 cells were arrested in S phase by AS-tDR-007333 overexpression, while inhibition of AS-tDR-007333 decreased the rate of S phase cells (Fig. 3E–H). In support of these, similar results were also observed in A549 cells (Fig. 3I–L). We then examined the invasive ability of AS-tDR-007333 in NSCLC cells using the transwell assay. We found that the number of migrated cells in the AS-tDR-007333-overexpression group was significantly higher than that of control group, while AS-tDR-007333 inhibitor reversed these effects (Fig. 3M–Q). However, no significant effects of AS-tDR-007333 or its inhibitor on apoptosis were observed in both PC9 and A549 cells (Additional file 2: Fig. S3a-3e).

### AS-tDR-007333 enhances NSCLC cell proliferation through up-regulating MED29

**Fig. 4:**
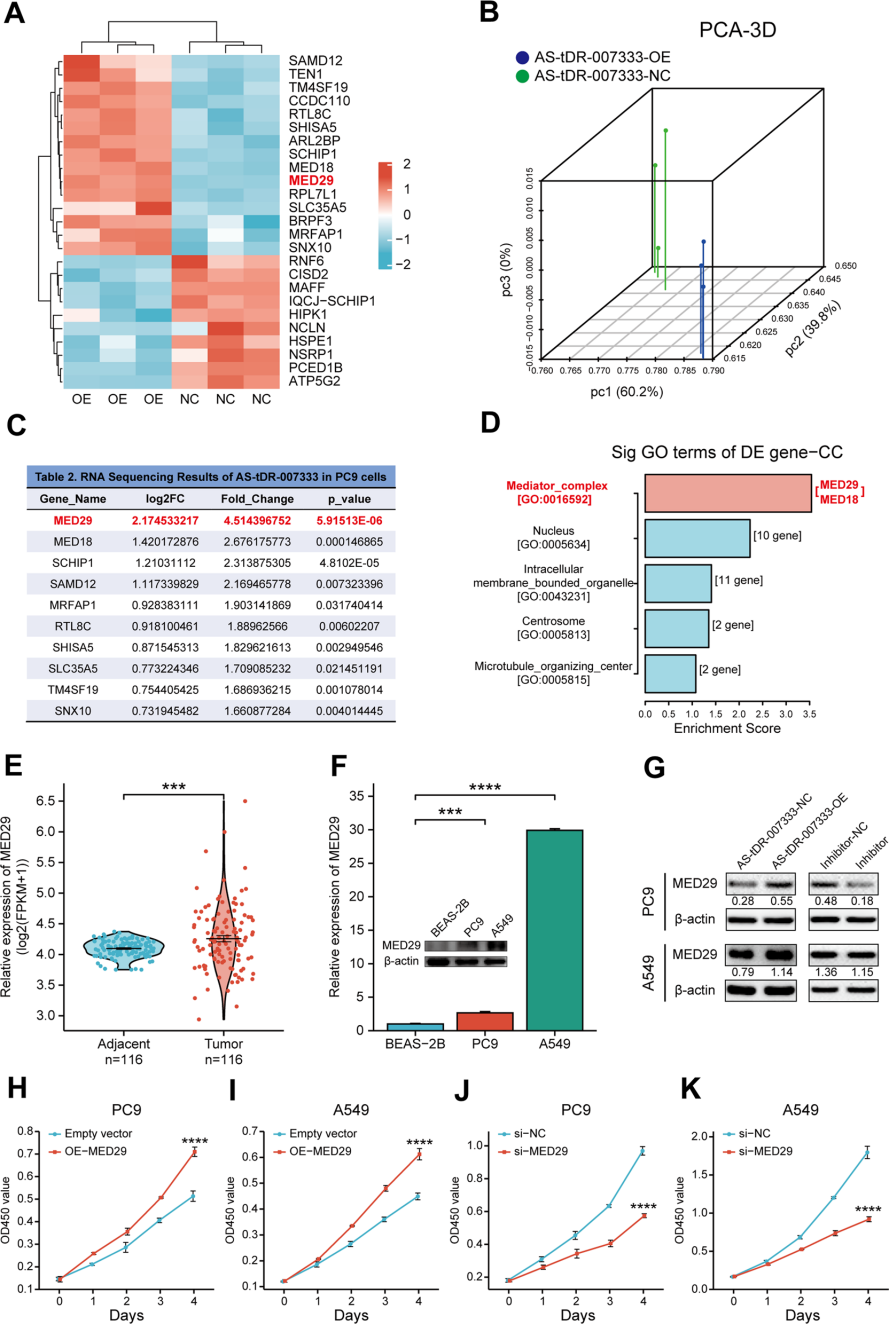
AS-tDR-007333 enhances NSCLC cell proliferation through up-regulating MED29. **A** RNA-seq revealed the differentially regulated genes between AS-tDR-007333-overexpression cells and control cells. **B** Principal component analysis (PCA) showed a clear difference between genetic components of AS-tDR-007333-overexpression cells and that of control cells. **C** Top ten differentially expressed genes regulated by AS-tDR-007333, with MED29 displaying the highest fold change and smallest *P* value. **D** Gene Ontology (GO) analysis revealed that AS-tDR-007333 was significantly correlated with mediator complex (MED) pathway genes. **E** MED29 gene was significantly up-regulated in NSCLC tumor tissues compared with that in adjacent tissues (analysis on TCGA data). **F** The expression levels of MED29 gene and protein in NSCLC cells were significantly higher than that in BEAS-2B cells. **G** Western blot analysis showed that overexpression of AS-tDR-007333 promoted MED29 protein expression in both PC9 and A549 cells, while knockdown of AS-tDR-007333 suppressed MED29 protein expression. Numerical numbers denoted the ratio of integrated optical density (IOD) of MED29/IOD of β-actin. **H**, **I** Up-regulation of MED29 promoted cell proliferation of NSCLC cells. **J**, **K** Knockdown of MED29 expression inhibited NSCLC cell proliferation. *P < 0.05; **P < 0.01; ***P < 0.001; ****P < 0.0001

To explore the target genes regulated by AS-tDR-007333, we transfected AS-tDR-007333 into NSCLC cells. RNA-seq analysis revealed that transcriptome profiles in AS-tDR-007333-overexpressed cells were distinct from that of NC cells (Fig. 4A, B, Additional file 1: Table S6). Among the differentially expressed genes, MED29 displayed the highest up-regulation by AS-tDR-007333 overexpression (Fig. 4C). Gene ontology (GO) analysis revealed that the differentially expressed genes were significantly enriched in mediator complex (MED) of cellular component (Fig. 4D; Additional file 1: Table S7). Gene set enrichment analysis (GSEA) also showed that MED pathway genes, particularly MED29, were enriched in AS-tDR-007333-overexpressed cells (Additional file 1: Table S8; Additional file 2: Fig. S4a-b). Furthermore, the expression levels of MED29 in NSCLC tumor tissues were significantly higher than that in the adjacent tissues (Fig. 4E, Additional file 2: Fig. S5a-f, S14c). Similarly, qRT-PCR assay and Western blot analyses verified that MED29 gene and protein were significantly up-regulated in NSCLC cells (Fig. 4F). Interestingly, the expression levels of AS-tDR-007333 were positively correlated that of MED29 in NSCLC tumor tissues (Additional file 2: Fig. S14f). Taken together, these results indicated that AS-tDR-007333-induced up-regulation of MED29 may play an important role in the pathogenesis of NSCLC.

To verify the relationship between AS-tDR-007333 and MED29 in NSCLC, we transfected AS-tDR-007333 into NSCLC cells. Western blot and qRT-PCR analyses showed that overexpression of AS-tDR-007333 significantly promoted MED29 expression, while inhibition of AS-tDR-007333 decreased the expression levels of MED29 (Fig. 4G; Additional file 2: Fig. S6a, S6b). Moreover, CCK-8 assays revealed that up-regulation of MED29 promoted cell proliferation in both A549 and PC9 cells (Fig. 4H, I), whereas knockdown of MED29 inhibited the growth rate of NSCLC cells (Fig. 4J, K). In addition, rescue assays showed that co-transfection of AS-tDR-007333 with si-MED29 into NSCLC cells resulted in significantly decreased of cell proliferation, compared with that of transfection of AS-tDR-007333 alone (Additional file 2: Fig. S6c, S6d), suggesting that the biological function of AS-tDR-007333 was partly dependent on MED29. Together, these results suggested that AS-tDR-007333 promoted the expression of MED29, which subsequently acted as an oncogene to enhance the proliferation of NSCLC cells.

### AS-tDR-007333 interacts with HSPB1 that epigenetically augments MED29 transcription

**Fig. 5:**
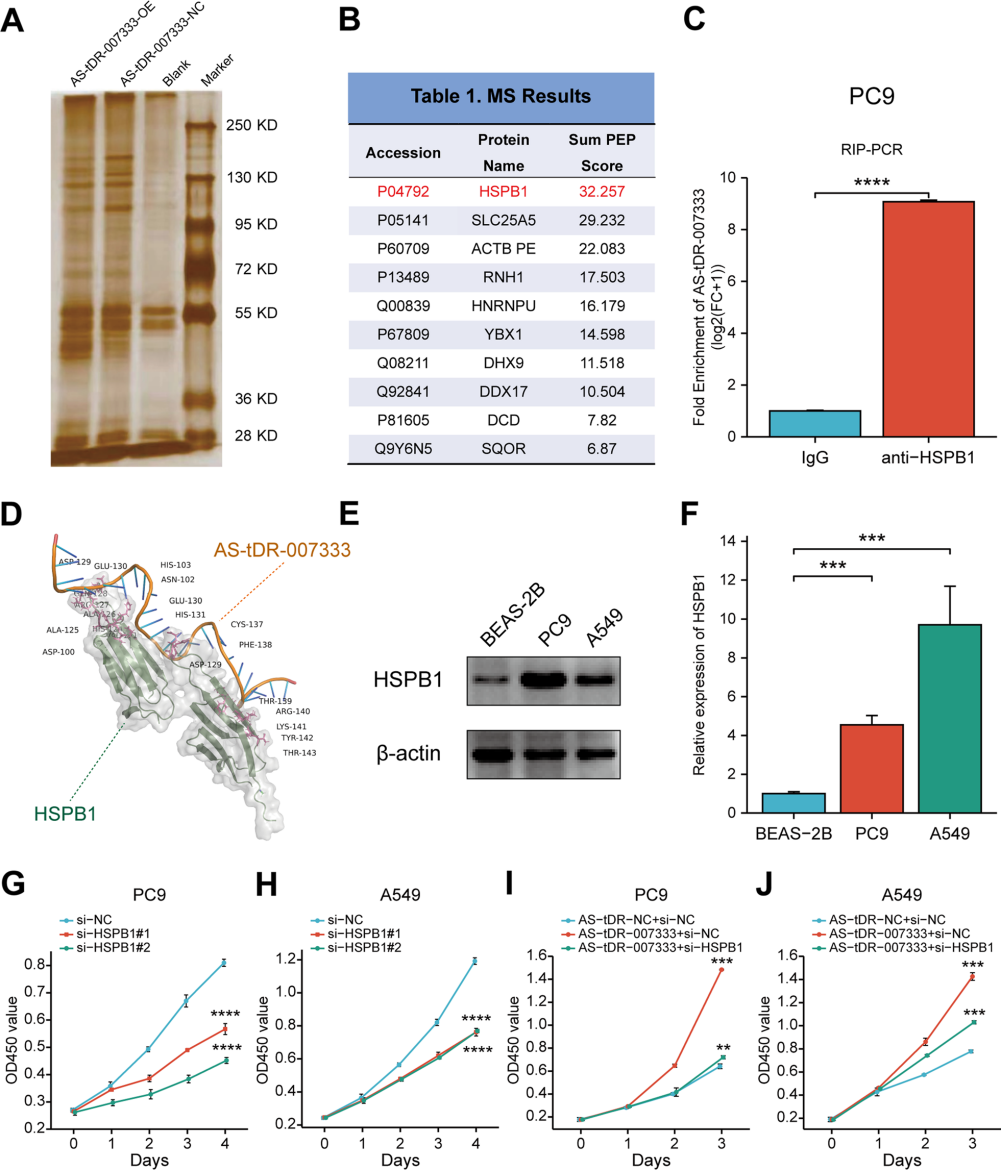
AS-tDR-007333 directly binds to and interacts with HSPB1 in NSCLC cells. **A** Silver SDS-PAGE gel image revealed proteins immunoprecipitated by AS-tDR-007333 and its antisense RNA in PC9 cells. **B** AS-tDR-007333 binding proteins with top matching scores identified by mass spectrometry. **C** RIP assay followed by qRT-PCR analysis confirmed that AS-tDR-007333 specifically bound to HSPB1 protein in PC9 cells. **D** Predicted 3D structure of the AS-tDR-007333–HSPB1complex. **E** The expression levels of HSPB1 protein in NSCLC cells were higher than that in BEAS-2B cells. **F** The expression levels of HSPB1 gene in NSCLC cells were significantly higher than that in BEAS-2B cells. **G** si-HSPB1 significantly repressed cell proliferation of PC9 cells. **H** Inhibition of HSPB1 gene expression decreased cell proliferation rate of A549 cells. **I** Rescue assays showed that co-transfection of AS-tDR-007333 and si-HSPB1 suppressed the promoting ability of AS-tDR-007333 on cell proliferation of PC9 cells. **J** Co-transfection of AS-tDR-007333 and si-HSPB1 decreased the capacity of AS-tDR-007333 for enhancing cell proliferation rate in A549 cells. *P < 0.05; **P < 0.01; ***P < 0.001

To elucidate the mechanism by which AS-tDR-007333 exerted its biological functions in NSCLC, we performed RNA pull-down experiment, followed by mass spectrometry analysis in NSCLC cells (Fig. 5A). We found that AS-tDR-007333 precipitated with several cancer-related RNA-binding proteins, including HSPB1, DHX9, ACTB, YBX3, and ILF2 (Fig. 5B). Among them, we were particularly interested in HSPB1, because HSPB1 had the highest matching score and has been reported to be associated with the development and progression of NSCLC [14, 15]. Moreover, HSPB1 was also found to be up-regulated in NSCLC and other types of cancers (Additional file 2: Fig. S7a-f, S14a) and was positively correlated with that of AS-tDR-007333 in NSCLC tumor tissues (Additional file 2: Fig. S14d). RIP assay using an antibody against HSPB1 confirmed that AS-tDR-007333 specifically bound to endogenous HSPB1 (Fig. 5C). The computational protein-RNA docking analysis (http://hdock.phys.hust.edu.cn/) also indicated that several amino acid residues in HSPB1 protein are critical for AS-tDR-007333 binding (Fig. 5D). Furthermore, CHX assay showed that AS-tDR-007333 did not affect the stability of HSPB1 proteins (Additional file 2: Fig. S8a-b). These data suggested that AS-tDR-007333 might exert its biological function by directly binding to HSPB1 in NSCLC cells.

To determine whether AS-tDR-007333 may regulate NSCLC cells proliferation through interacting with HSPB1. We first examined the expression levels of HSPB1 in NSCLC cell lines and BEAS-2B cells. Both qRT-PCR and Western blot assays consistently showed that the expression levels of HSPB1 in NSCLC cells were significantly higher than that of BEAS-2B cells (Fig. 5E, F). Rescue experiments by co-transfection of si-HSPB1 and AS-tDR-007333 into NSCLC cells revealed that the increased cell proliferation capacity by AS-tDR-007333 overexpression could be significantly diminished by si-HSPB1 (Fig. 5G–J), indicating that the effect of AS-tDR-007333 on NSCLC cell proliferation was functionally dependent, at least in part, on HSPB1.

**Fig. 6:**
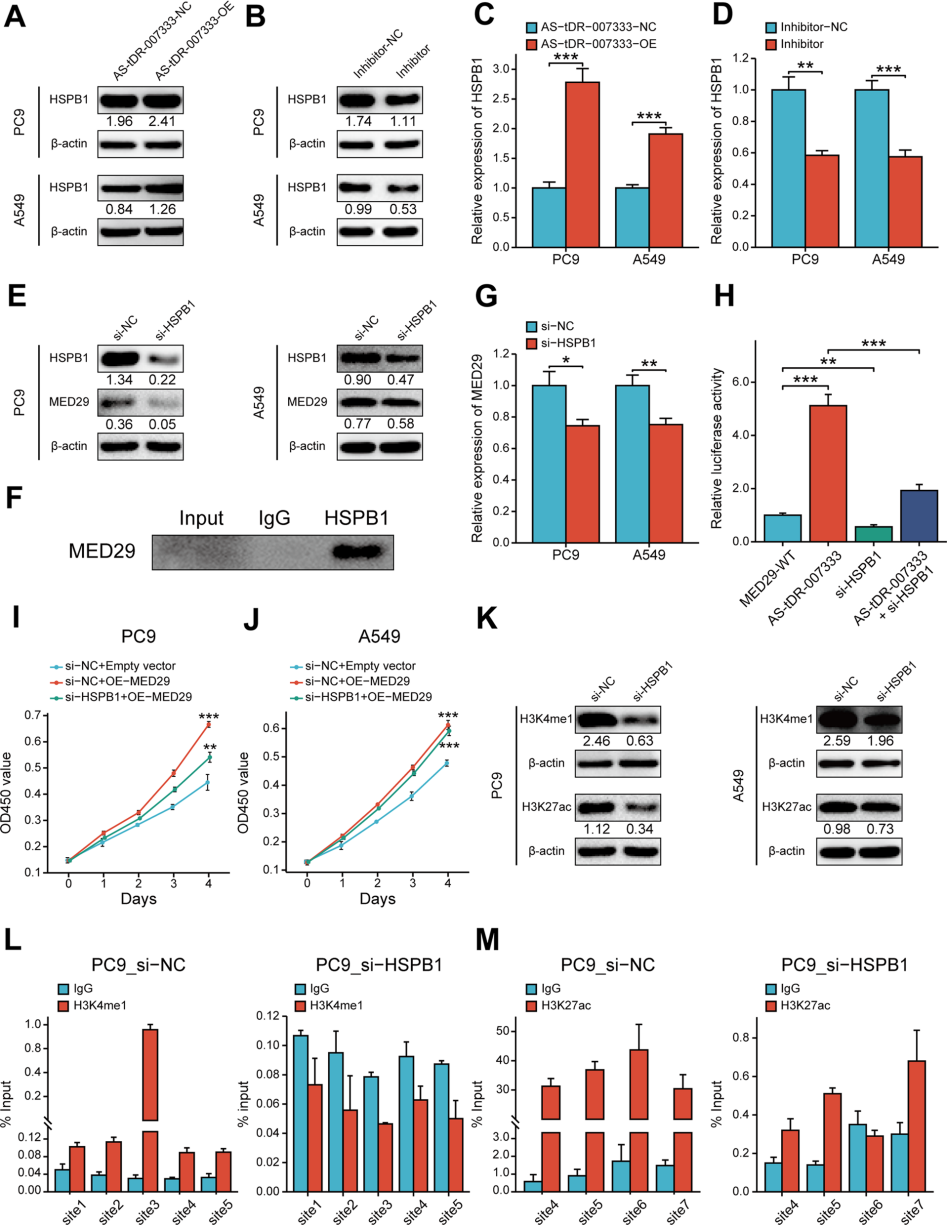
AS-tDR-007333 activates HSPB1 to regulate H3K4me1 and H3K27ac levels in MED29 promoter region. **A** AS-tDR-007333 up-regulated HSPB1 protein expression in NSCLC cells. Numerical numbers denoted the ratio of integrated optical density (IOD)/IOD of β-actin. **B** Blocking AS-tDR-007333 suppressed HSPB1 protein expression. **C** Up-regulation of AS-tDR-007333 promoted HSPB1 gene expression in NSCLC cells. **D** Knockdown of AS-tDR-007333 decreased HSPB1 gene expression in NSCLC cells. **E** si-HSPB1 inhibited both HSPB1 and MED29 protein expressions. **F** Co-IP assay indicated that HSPB1 protein interacted with MED29 protein in A549 cells. **G** Inhibition of HSPB1 suppressed MED29 gene expression in PC9 and A549 cells. **H** Rescue assays showed an interaction between AS-tDR-007333 and si-HSPB1 for regulating MED29 promoter activity. **I**, **J** Co-transfection of MED29 and si-HSPB1 into NSCLC cells rescued the promoting effect of MED29 on cell proliferation. **K** Western blot analysis revealed that knockdown of HSPB1 suppressed the expression of H3K4me1 and H3K27ac in NSCLC cells. **L** ChIP-qPCR results of H3K4me1 signal on different regions of MED29 promoter in the presence or absence of si-HSPB1. **M** ChIP-qPCR results of H3K27ac expression levels on different regions of MED29 promoter in the presence or absence of si-HSPB1. *P < 0.05; **P < 0.01; ***P < 0.001

As AS-tDR-007333 could interact with HSPB1 and enhance MED29 expression, we hypothesized that AS-tDR-007333 may exert its biological function by regulating HSPB1, which further modify the expression and function of MED29. To test our hypothesis, we transfected AS-tDR-007333 and its inhibitor into NSCLC cells and found that overexpression of AS-tDR-007333 increased the expression levels of HSPB1 gene and protein, whereas knockdown of AS-tDR-007333 decreased HSPB1 expressions (Fig. 6A–D). Interestingly, knockdown HSPB1 not only suppressed the expression of HSPB1, but also inhibited MED29 expression (Fig. 6E, G). Co-IP assay showed that HSPB1 could bind to MED29 in NSCLC cells (Fig. 6F), suggesting a potential interaction between HSPB1 and MED29. Rescue experiments using luciferase assay verified that up-regulation of AS-tDR-007333 significantly increased the promoter activity of MED29, whereas co-transfection of AS-tDR-007333 with si-HSPB1 diminished MED29 promoter activity (Fig. 6H). Functionally, co-transfection of MED29 and si-HSPB1 into NSCLC cells significantly repressed the effect of MED29 on cell proliferation (Fig. 6I, J). Taken together, our results indicated that AS-tDR-007333 may enhance NSCLC cell proliferation through activating the HSPB1-MED29 interactions.

Histone modification plays a vital role in epigenetic regulation of gene transcription [16]. As the UCSC genome browser showed that the MED29 promoter region contains H3K4me1 and H3K27ac, histone marks for transcription activation (Additional file 2: Fig. S11a), we examined whether HSPB1 expression may affect histone marks expression in MED29 promoter. JASPAR database analysis predicted that there were several putative binding sites in the MED29 promoter region for HSPB1 (Additional file 2: Fig. S11b). Western blot assay showed that si-HSPB1 suppressed the expression levels of H3K4me1 and H3K27ac compared to that in wild-type cells (Fig. 6K). Further, ChIP-qPCR assay demonstrated that knockdown of HSPB1 significantly decreased H3K4me1 and H3K27ac levels in MED29 promoter region (Fig. 6L, M). Thus, these data indicated that AS-tDR-007333 may promote cancer cell proliferation, at least in part, through HSPB1-mediated H3K4me1 and H3K27ac modifications in the promoter of MED29.

### AS-tDR-00733 up-regulates MED29 via activating ELK4-mediated transcriptional regulation

**Fig. 7:**
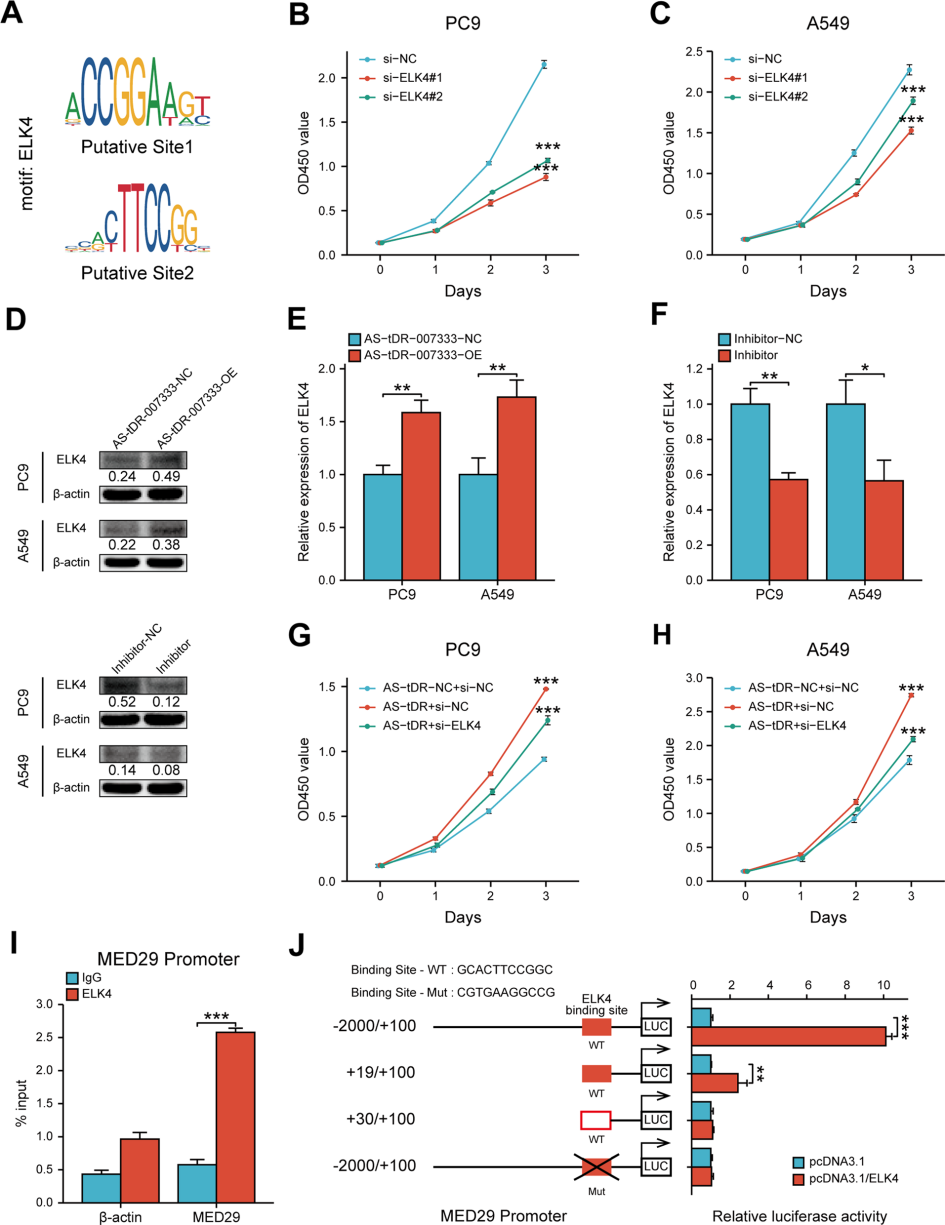
AS-tDR-00733 regulates MED29 through ELK4-mediated transcriptional activation. **A** Schematic view of the putative binding sites and sequences of ELK4 on MED29 promoter region, predicted by the JASPAR and UCSC websites. **B** Inhibitive effect of si-ELK4 on PC9 cell proliferation. **C** The impact of ELK4 knockdown by si-ELK4 on A549 cell proliferation. **D** AS-tDR-007333 increased ELK4 protein expression levels in A549 and PC9 cells (upper); AS-tDR-007333-inhibitor repressed the expression of ELK4 protein in NSCLC cells (lower). **E** Up-regulation of AS-tDR-007333 promoted the expression of ELK4 gene in NSCLC cells. **F** AS-tDR-007333-inhibitor suppressed ELK4 gene expression in NSCLC cells. **G** Interaction effects between si-ELK4 and AS-tDR-007333 on PC9 cell proliferation. **H** Co-transfection of si-ELK4 and AS-tDR-007333 inhibited the promoting effect of AS-tDR-007333 on A549 cell proliferation. **I** ChIP-PCR assay verified the reliability of the binding sequences of ELK4 on MED29 promoter region. **J** Luciferase reporter gene assays showed that binding of ELK4 to the wild-type MED29 promoter significantly increased the transcription of MED29. *P < 0.05; **P < 0.01; ***P < 0.001

Since HSPB1-MED29 interaction could only partly explain MED29 expression, we speculated that there may be additional mechanisms in AS-tDR-007333-associated regulation of MED29 expression. Because it has been proposed that transcription factor (TF) could target specific MED subunit to induce transcriptional responses [17], we reasoned that MED29 transcription may be affected by particular transcriptional factor. Using JASPAR and UCSC database analyses, we found that MED29 promoter contained putative binding sites for transcription factor ELK4 (Fig. 7A). Indeed, ELK4 was found to be up-regulated in NSCLC cells and in NSCLC tissues (Additional file 2: Fig. S9a-c, S10a-b, S14b) and its expression levels were positively correlated with that of AS-tDR-007333 in NSCLC tumor tissues (Additional file 2: Fig. S14e). To assess the impact of ELK4 on NSCLC, we transfected si-ELK4 into NSCLC cells and found si-ELK4 significantly suppressed NSCLC proliferation (Fig. 7B, C). To determine whether AS-tDR-007333 may influence ELK4 expression, we transfected AS-tDR-007333 into NSCLC cells. We found that overexpression of AS-tDR-007333 significantly promoted the expression levels of ELK4 in NSCLC cells (Fig. 7D, E); in contrast, inhibition of AS-tDR-007333 significantly decreased ELK4 expression (Fig. 7D, F). Rescue experiments further confirmed that AS-tDR-007333 was functionally interacted with si-ELK4 in NSCLC cell proliferation (Fig. 7G, H). To investigate whether ELK4 may directly affect MED29 expression, we performed ChIP-PCR assays, which confirmed that ELK4 directly bound to the predicted promoter regions of MED29 gene (Fig. 7I). Luciferase reporter assay showed that overexpression of ELK4 significantly increased luciferase activity of the reporters containing the wild-type binding sites, compared to that of NC-vector cells (Fig. 7J). However, no significant change of the luciferase activity was observed on the binding of ELK4 with the mutant promoter of MED29 (Fig. 7J). Collectively, these findings suggested that AS-tDR-007333 interacted with ELK4 to modify MED29 promoter transcription.

### Targeting AS-tDR-007333 suppresses NSCLC cell growth in vivo

**Fig. 8:**
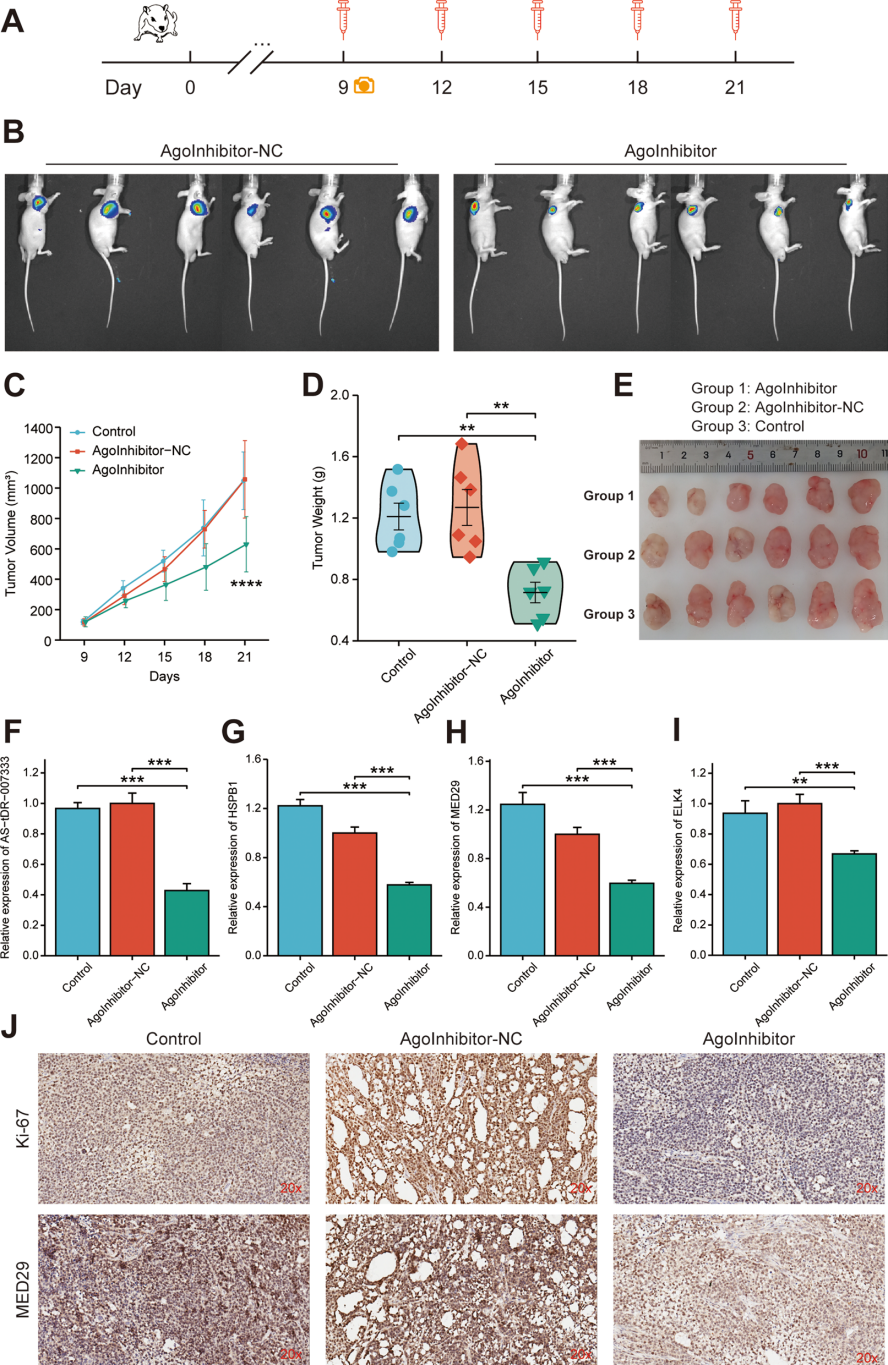
Targeting AS-tDR-007333 with inhibitor diminished NSCLC tumor growth in vivo. **A** Schematic experimental overview of the in vivo study. **B** Representative bioluminescence images of the xenograft tumors in mice after injecting with inhibitor, or inhibitor-NC, respectively. **C** The mean tumor volumes of AS-tDR-007333-inhibitor group were significantly smaller than that of NC group. **D** The mean tumor weight of the inhibitor group was lower than that of NC group. **E** Representative images of the xenograft tumors isolated from the three indicated groups. **F**–**I** qRT-PCR analysis showed that AS-tDR-007333-inhibitor suppressed the expression of AS-tDR-007333, MED29, ELK4, and HSPB1 in xenograft tumor tissues. **J** Representative images of IHC staining, indicating that AS-tDR-007333-inhibitor repressed expression levels of Ki-67 and MED29 proteins in xenograft tumors. *P < 0.05; **P < 0.01; ***P < 0.001; ****P < 0.0001

Given that AS-tDR-007333 acted as an oncogenic tRF in NSCLC, we hypothesized that inhibition of AS-tDR-007333 may have therapeutic effect on NSCLC. To evaluate the therapeutic efficacy of AS-tDR-007333-inhibitor in vivo (Fig. 8A), we synthesized AS-tDR-007333-targeting inhibitor with modification optimized for in vivo study. As shown in Fig. 8B, C, the tumor volume was significantly smaller in AS-tDR-007333-inhibitor group than that in NC or blank control groups during the entire experimental period, but no differences in body weight were observed among experimental groups (Additional file 2: Fig. S13). Moreover, the average tumor weight in AS-tDR-007333-inhibitor group was significantly (*P* < 0.01) lower than that of control groups (Fig. 8D, E). Furthermore, the expression levels of AS-tDR-007333, HSPB1, ELK4, and MED29 in xenograft tumor tissues were significantly suppressed in the AS-tDR-007333-inhibitor group compared with that of NC and control groups (Fig. 8F–I). In addition, administration of AS-tDR-007333-inhibitor also suppressed the expression levels of both MED29 and Ki-67 proteins in the xenograft tumor tissues (Fig. 8J). Thus, these findings suggest that AS-tDR-007333 inhibitor could suppress NSCLC tumor growth through inhibiting MED29 expression in vivo.

## Discussion

Through systematic profiling of tRFs in pre- and post-operation plasma from patients with NSCLC, we uncovered a new tRF named AS-tDR-007333, which is derived from the D-loop of tRNA^Gly−GCC^. We found that AS-tDR-007333 was up-regulated in NSCLC. Its increased levels were associated with worse prognosis of NSCLC patients and were able to discriminate NSCLC patients from healthy controls. We have also demonstrated that up-regulation of AS-tDR-007333 significantly promoted NSCLC malignancy, while inhibition of AS-tDR-007333 effectively suppressed NSCLC cell growth in vitro and in vivo. Our findings indicated that AS-tDR-007333 was an oncogenic tRF that had potential to serve as diagnostic and prognostic markers or therapeutic target for NSCLC.

The molecular mechanisms by which tRFs exert their functions are largely unclear. Prior studies have shown that some tRFs can directly bind to the 3′ untranslated regions (3′ UTRs) of target mRNAs, leading to translation suppression [18, 19]. Other tRFs repressed the stability of oncogenes by replacing the 3′-UTR of mRNAs. For instance, hypoxia-induced i-tRFs could decrease the stability of some oncogenes via YBX1 replacement [10]. tRFs could also bind to mRNA 3′-UTR and induce its degradation, resulting in decreased protein production [12]. More recently, it was reported that certain tRFs could bind to proteins and alter the phosphorylation status and the function of the target protein [20]. The present study demonstrated, for the first time to our knowledge, that tRFs (i.e., AS-tDR-007333) interacted with binding protein to modify histone modifications and activate transcription factor to enhance promoter activity, resulting in gene expression alteration. These findings expand our knowledge on the regulatory roles of tRFs in cancer cells.

We found that AS-tDR-007333 directly bound to HSPB1 with high specificity in NSCLC cells. The HSPB1 (HSP27) is a member of the highly conserved heat shock proteins (HSPs) which are expressed at low levels under normal conditions, but induced in response to cellular stresses, including heat shock, hypoxia, genotoxic agents, and overexpression of oncoproteins [21]. Previous studies showed that HSPB1 was highly expressed in a variety of human cancers, including lung cancer [22]. Overexpression of HSPB1 was associated with malignant properties of cancer cells, resistance to chemotherapy or radiotherapy, and poor prognosis [14, 23]. HSPB1 also increased cell proliferation by facilitating cell cycle progression [24], promoting migration and invasion [25], maintaining cancer stem cells [26], or inactivating the Hippo tumor suppressor pathway [27]. Nevertheless, the molecular mechanisms governing HSPB1 expression were unclear. Here, we showed that HSPB1 bound to and activated by AS-tDR-007333. Rescue assays showed that the impact of AS-tDR-007333 on cell proliferation is dependent, at least in part, on HSPB1. These results revealed a novel mechanism by which tRF interacted with HSPB1 to regulate NSCLC cell proliferation.

To elucidate the molecular mechanisms underlying AS-tDR-007333 functions in NSCLC, we examined AS-tDR-007333-induced transcriptome changes, leading to the identification of MED29 as the key target gene of AS-tDR-00733. The MED29 (previously known as IXL) is a subunit of regulatory Mediator (MED) complex and locates in the head module of the MED complex [28]. The MED complex stimulates the assembly of a pre-initiation complex (PIC) and recruitment of RNA Polymerase II (Pol II) to gene promoter to initiate gene expression [29]. While studies have showed that loss of MED function resulted in decreased mRNA levels and concomitant diminished expression of Pol II transcribed genes [30, 31], relatively little is known about the role of MED29 in cancers. There was only one report showing that MED29 was overexpressed in pancreatic cancer and promoted pancreatic cancer cell viability [32]. The present study shed light on the biological functions of MED29 in several ways. First, we showed that the expression of MED29 was up-regulated by tRF. Second, overexpression of MED29 promoted NSCLC cell proliferation. Third, down-regulation of AS-tDR-00733 was correlated with decreased expression of MED29 in vitro and in vivo. Collectively, these findings indicated that MED29 may function as an oncogene in tRF-mediated malignancy in NSCLC cells.

Little is known about the regulatory mechanisms that control MED29 expression. Here, we showed that AS-tDR-007333 stimulated MED29 expression via HSPB1-mediated histone modifications in MED29 promoter. Several studies have reported the impact of HSPB1 on gene expression [21]. It was reported that inhibition of histone deacetylase 6 (HDAC6) suppressed HSPB1-related biological processes [33]. But whether HSPB1 may regulate gene expression through modifying histone modifications is unclear. Histone modification is crucial in chromatin structure change which regulates the DNA replication and gene expression [34]. Among the many types of histone modifications, lysine methylation deregulation is the most important in carcinogenesis [35]. Monomethylation on lysine 4 of histone H3 (H3K4me1) is mainly found in enhancer regions of the chromatin, and multiple studies have shown that enhancer usage is changed in cancer cells favoring the expression of growth-associated genes [36]. H3K4me1 also exists at promoter regions proximal to transcription start sites [37]. H3K27ac is an epigenetic mark of active enhancers. Elevation of H3K27ac on the enhancer and promoter regions promoted gene transcription [38]. But lack of H3K27ac resulted in decreased expression of the proximal genes of enhancers [39]. Coexistence of H3K27ac and H3K4me1 was associated with active enhancers [40]. In this study, we found that AS-tDR-007333 bound to and interacted with HSPB1. ChIP assay revealed that H3K4me1 and H3K27ac were enriched in the promoter region of MED29. Knockdown of HSPB1 decreased the H3K4me1 and H3K27ac levels around MED29 promoter region. These observations suggested an essential role of AS-tDR-007333-HSPB1 complex in modifying histone modifications at MED29 promoter. Whether AS-tDR-007333 may bind to other transcriptional regulators for regulating H3K4me1 and H3K27ac levels warrants further investigations.

In addition to the influence of AS-tDR-007333-HSPB1-histone modification axis on MED29 gene expression, we also found that AS-tDR-007333 stimulated the expression of ELK4 to enhance the activity of MED29 promoter. ELK4 is a transcription factor belonging to the ternary complex factor subfamily of E twenty-six domain transcription genes [41]. ELK4 has been identified as a proto-oncogene whose overexpression was associated with the malignant phenotypes of prostate, melanoma, and gastric cancers [42]. ELK4 is also involved in immune regulation by directing differentiation programs in αβ CD8 + T cells [43]. But the target gene of ELK4 has not been characterized. Here, we showed that AS-tDR-007333 promoted ELK4 expression. Moreover, we demonstrated that ELK4 directly bound to the promoter of MED29. Furthermore, we revealed that overexpression of ELK4 enhanced the activity of MED29 promoter and increased its transcription. Therefore, our results indicated that AS-tDR-007333 could regulate MED29 expression via both epigenetic and transcriptional pathways.

Since AS-tDR-007333 was identified to be an oncogenic tRF and knockdown of AS-tDR-007333 significantly inhibited NSCLC cell malignancy in vitro, we explored whether targeting AS-tDR-007333 might have therapeutic effect on NSCLC tumor growth in vivo. In agreement with the findings of in vitro experiments, our in vivo study demonstrated that AS-tDR-007333-inhibitor effectively repressed NSCLC cell growth in animal models. Interestingly, AS-tDR-007333-inhibitor also suppressed the expressions level of MED29 protein in xenograft tumor, suggesting that the tumor suppression function of AS-tDR-007333-inhibitor was associated with down-regulation of MED29 expression. Taken together, these data strongly support our hypothesis that AS-tDR-007333 may be a potential therapeutic target for NSCLC treatment.

## Conclusions

**Fig. 9:**
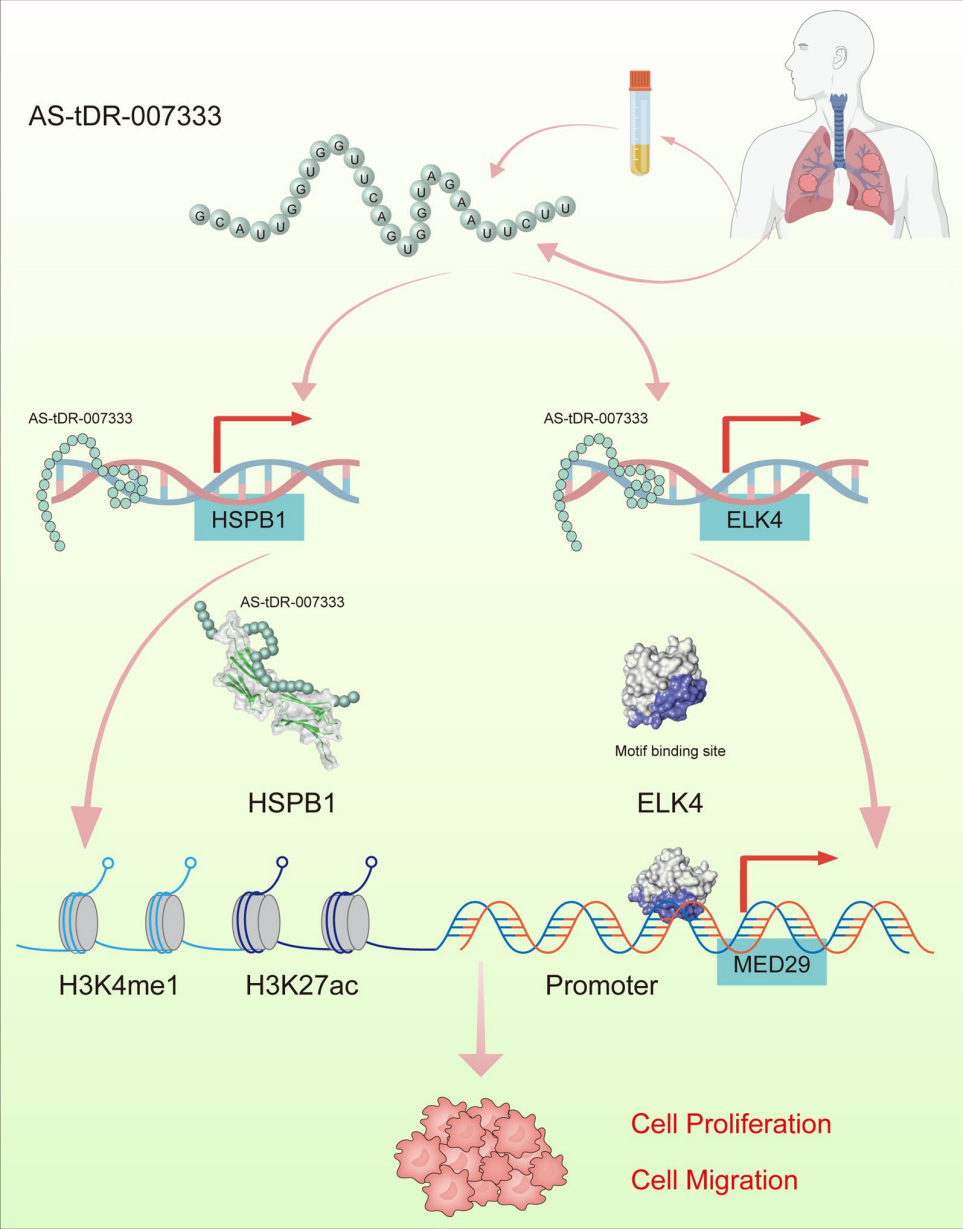
The schematic diagram shows how AS-tDR-007333 promotes tumorigenesis of NSCLC through the HSPB1-MED29 and ELK4-MED29 axes

In summary, we identified AS-tDR-007333 as a novel oncogenic tRF in NSCLC. We revealed that AS-tDR-007333 could promote the malignancy of NSCLC cells by targeting at oncogenic MED29 through activating HSPB1- and ELK4-mediated epigenetic and transcriptional regulation axes. We also demonstrated that inhibition of AS-tDR-007333 suppressed NSCLC cell proliferation in vitro and in vivo. Our data highlight the importance of tRF in NSCLC and suggest that AS-tDR-007333 can act as a promising diagnostic/prognostic biomarker and new therapeutic target for NSCLC (Fig. 9).

## Supplementary Information

**Additional file 1: Table S1.** Characteristics of NSCLC patients recruited for tRF and tiRNA sequencing. **Table S2.** Sequences of primers, inhibitor, and probes used in this study. **Table S3.** Expression levels of cytoplasmic AS-tDR-007333 between NSCLC tumor and adjacent tissues. **Table S4.** Expression levels of nucleus AS-tDR-007333 between NSCLC tumor and adjacent tissues. **Table S5.** Cox regression analysis on the association of AS-tDR-007333 with NSCLC prognosis. **Table S6.** Genes significantly regulated by AS-tDR-007333 over expression. **Table S7.** Gene ontology enrichment analysis of up-regulated genes by AS-tDR-007333. **Table S8.** Gene set enrichment analysis in AS-tDR-007333-overexpression cells vs. control cells.

**Additional file 2: Figure S1.** Characteristics of AS-tDR-007333. **Figure S2.** The transfection efficiencies of mimics, plasmids, and si-RNAs in NSCLC cells. **Figure S3.** AS-tDR-007333 did not affect apoptosis phenotypes in NSCLC cells. **Figure S4.** Gene set enrichment analysis (GSEA) of AS-tDR-007333-treated cells. **Figure S5.** In silico analysis of MED29 in NSCLC based on TCGA database. **Figure S6**. AS-tDR-007333 regulates MED29 expression and functionally interacts with MED29 in NSCLC cells. **Figure S7.** HSPB1 is up-regulated in NSCLC (in silico analysis based on TCGA database). **Figure S8.** CHX-chase assay results suggested that AS-tDR-007333 may not affect HSPB1 protein degradation. **Figure S9.** ELK4 was up-regulated in NSCLC based on TCGA database. **Figure S10**. ELK4 was up-regulated in NSCLC cells. **Figure S11.** Schematic diagram of genomic organization and chromatin state of the human MED29 gene locus. **Figure S12.** Overview of AS-tDR-007333 staining in tissue microarrays (TMAs) spots. **Figure S13.** AS-tDR-007333 inhibitor did not affect the body weight different subgroups of rats during the period of experiments. **Figure S14.** Correlations between AS-tDR-007333 and HSPB1, ELK4, and MED29 in NSCLC tumor tissues.

## Data Availability

The datasets used and analyzed during the current study are available from the corresponding author on reasonable request. tRF-sequencing and RNA-seq data have been deposited in the NCBI GEO database with the accession number GSE185546 and GSE184690.

## Abbreviations

tRFs: Transfer RNA-derived fragments
NSCLC: Non-small cell lung cancer
qRT-PCR: Quantitative real-time polymerase chain reaction
ROC curve: Receiver operating characteristic curve
OS: Overall survival
RNA-seq: RNA-sequencing
GO: Gene ontology
GSEA: Gene set enrichment analysis
MED: Mediator complex
TCGA: The Cancer Genome Atlas
TMA: Tissue microarray
IHC: Immunohistochemistry
FISH: Fluorescence in situ hybridization
3′UTR: 3′-Untranslated region
RIP: RNA immunoprecipitation
si-RNA: Small interfering-RNA
ChIP: Chromatin immunoprecipitation
