# Supplementary material for: A novel tRNA-derived fragment AS-tDR-007333 promotes the malignancy of NSCLC via the HSPB1/MED29 and ELK4/MED29 axes
Source: J Hematol Oncol. 2022 May 7;15:53. doi: 10.1186/s13045-022-01270-y (PMC9077895; doi:10.1186/s13045-022-01270-y)
Supplement: Supplementary file 1 — Additional file 1: Table S1. Characteristics of NSCLC patients recruited for tRF and tiRNA sequencing. Table S2. Sequences of primers, inhibitor, and probes used in this study. Table S3. Expression levels of cytoplasmic AS-tDR-007333 between NSCLC tumor and adjacent tissues. Table S4. Expression levels of nucleus AS-tDR-007333 between NSCLC tumor and adjacent tissues. Table S5. Cox regression analysis on the association of AS-tDR-007333 with NSCLC prognosis. Table S6. Genes significantly regulated by AS-tDR-007333 over expression. Table S7. Gene ontology enrichment analysis of up-regulated genes by AS-tDR-007333. Table S8. Gene set enrichment analysis in AS-tDR-007333-overexpression cells vs. control cells. [file 13045_2022_1270_MOESM1_ESM.zip › 13045_2022_1270_MOESM1_ESM/Table S2..docx]

| **Table S2**. Sequences of synthesized tRF, tRF inhibitor, primers, and probes used in this study | |
| --- | --- |
| **Oligo names** | **Sequences (5 ́-3 ́)** |
| AS-tDR-007333 | GCAUUGGUGGUUCAGUGGUAGAAUUCUU |
| AS-tDR-007333-NC | GAGAAUUUUGAUGGCCGUUCUGUAUGUG |
| AS-tDR-007333 Inhibitor | AAGAAUUCUACCACUGAACCACCAAUGC |
| AS-tDR-007333 Inhibitor-NC | CACAUACAGAACGGCCAUCAAAAUUCUC |
| AgoInhibitor | AAGAAUUCUACCACUGAACCACCAAUGC |
| AgoInhibitor-NC | CACAUACAGAACGGCCAUCAAAAUUCUC |
| FISH Probe for AS-tDR-007333 | AAGAATTCTACCACTGAACCACCAATGC |
| Primers for AS-tDR-007333  (Ribobio) | Bulge-LoopTM h-tRFs-RT Primer: #8003848  Bulge-LoopTM h-tRFs-Forward Primer: #8003849  Bulge-LoopTM h-tRFs-Reverse Primer: #ssD089261711 |
| Primers for U6  (Ribobio) | Bulge-LoopTM U6-RT Primer: #ssD0904071008  Bulge-LoopTM U6-Forward Primer: #ssD0904071006  Bulge-LoopTM U6-Reverse Primer: #ssD0904071007 |
| Primers for HSPB1 | Forward: CAGAGCAGAGTCAGCCAGCA  Reverse: TGGTCGAAGAGGCGGCTAT |
| Primers for MED29 | Forward: CTGGTGTATCGGGTCCTAGTTC  Reverse: GGGATGAGCATCTTATAACGCT |
| Primers for ELK4 | Forward: ACCACCCATTTCGTCCATACC  Reverse: CTGGAAGTTCCATTGGCTGAG |
| Primers for site +19 ~ +29 in MED29-promoter | Forward: GCGGGACTAACTAGCAAACGG  Reverse: GCTGGGATGCAGCCATCTT |
| Primers for site 1 in MED29-promoter | Forward: TACTGGACGAACCTGGGTGG  Reverse: GCTCAATTGTTAATGGAGCAAGC |
| Primers for site 2 in MED29-promoter | Forward: CTGAGCAGACAGCAGCCAAC  Reverse: TCAAGTACTGCAATAGCCTCCC |
| Primers for site 3 in MED29-promoter | Forward: GGAACATTAGTGGCTCAAAAGTG  Reverse: CTGTCAATCCCGTCCTCTCC |
| Primers for site 4 in MED29-promoter | Forward: AACCTTTCAAAAGGTAGGAGGG  Reverse: AAAGGGATTGAGCACTTGGC |
| Primers for site 5 in MED29-promoter | Forward: ATGTACTCAATCGTCTTCGGAAG  Reverse: ACGTCATGAAGCGCTCACC |
| Primers for site 6 in MED29-promoter | Forward: CTGGTGACGTTGTTCAGCAGA  Reverse: CACCTCATCTCAACCCACTTTC |
| Primers for site 7 in MED29-promoter | Forward: TGATCCGAGGAAGGCCCA  Reverse: AATCCGTGTGTGGTTCCGA |
| Primers for GAPDH | Forward: GAACGGGAAGCTCACTGG  Reverse: GCCTGCTTCACCACCTTCT |
| si-HSPB1#1 | CGGACGAGCTGACGGTCAA |
| si-HSPB1#2 | GCGTGTCCCTGGATGTCAA |
| si-MED29#1 | GAGCAGTGATGGACCCATA |
| si-MED29#2 | GTCACAGAGTTGTGACAGT |
| si-ELK4#1 | GCCGCAATGACTACATACA |
| si-ELK4#2 | CTCCTCCAATGTAAAGCTT |
| si-NC  (Ribobio) | # siN0000001-1 |
