# Supplementary material for: A novel tRNA-derived fragment AS-tDR-007333 promotes the malignancy of NSCLC via the HSPB1/MED29 and ELK4/MED29 axes
Source: J Hematol Oncol. 2022 May 7;15:53. doi: 10.1186/s13045-022-01270-y (PMC9077895; doi:10.1186/s13045-022-01270-y)
Supplement: Supplementary file 1 — Additional file 1: Table S1. Characteristics of NSCLC patients recruited for tRF and tiRNA sequencing. Table S2. Sequences of primers, inhibitor, and probes used in this study. Table S3. Expression levels of cytoplasmic AS-tDR-007333 between NSCLC tumor and adjacent tissues. Table S4. Expression levels of nucleus AS-tDR-007333 between NSCLC tumor and adjacent tissues. Table S5. Cox regression analysis on the association of AS-tDR-007333 with NSCLC prognosis. Table S6. Genes significantly regulated by AS-tDR-007333 over expression. Table S7. Gene ontology enrichment analysis of up-regulated genes by AS-tDR-007333. Table S8. Gene set enrichment analysis in AS-tDR-007333-overexpression cells vs. control cells. [file 13045_2022_1270_MOESM1_ESM.zip › 13045_2022_1270_MOESM1_ESM/Table S7..docx]

| **Table S7.** Gene ontology enrichment (CC) analysis of genes up-regulated by AS-tDR-007333-overexpression | | | | | | | | |
| --- | --- | --- | --- | --- | --- | --- | --- | --- |
| **ID** | **Term** | **P-value** | **FDR** | **Enrichment**  **Score** | **Fold**  **Enrichment** | | **Gene Ratio** | **Genes** |
| **GO:0016592** | **Mediator complex** | **0.0003** | **0.0231** | **3.5403** | **78.4413** | **0.1538** | | **MED29, MED18** |
| GO:0005634 | Nucleus | 0.0059 | 0.0803 | 2.2317 | 1.9890 | 0.7692 | | ARL2BP, BRPF3, CCDC110, MED18, MED29, MRFAP1, RPL7L1, SHISA5, SNX10, TEN1, |
| GO:0043231 | Intracellular membrane-  Bounded organelle | 0.0389 | 0.2790 | 1.4097 | 1.4758 | 0.8461 | | ARL2BP, BRPF3, CCDC110, MED18, MED29, MRFAP1, RPL7L1, SHISA5, SLC35A5, SNX10, TEN1 |
| GO:0005813 | centrosome | 0.0444 | 0.2790 | 1.3529 | 5.8561 | 0.1538 | | ARL2BP, SNX10 |
| GO:0005815 | Microtubule organizing  center | 0.0838 | 0.3194 | 1.0766 | 4.0888 | 0.1538 | | ARL2BP//SNX10 |
