# Supplementary figures and images for: A novel tRNA-derived fragment AS-tDR-007333 promotes the malignancy of NSCLC via the HSPB1/MED29 and ELK4/MED29 axes
Source: J Hematol Oncol. 2022 May 7;15:53. doi: 10.1186/s13045-022-01270-y (PMC9077895; doi:10.1186/s13045-022-01270-y)

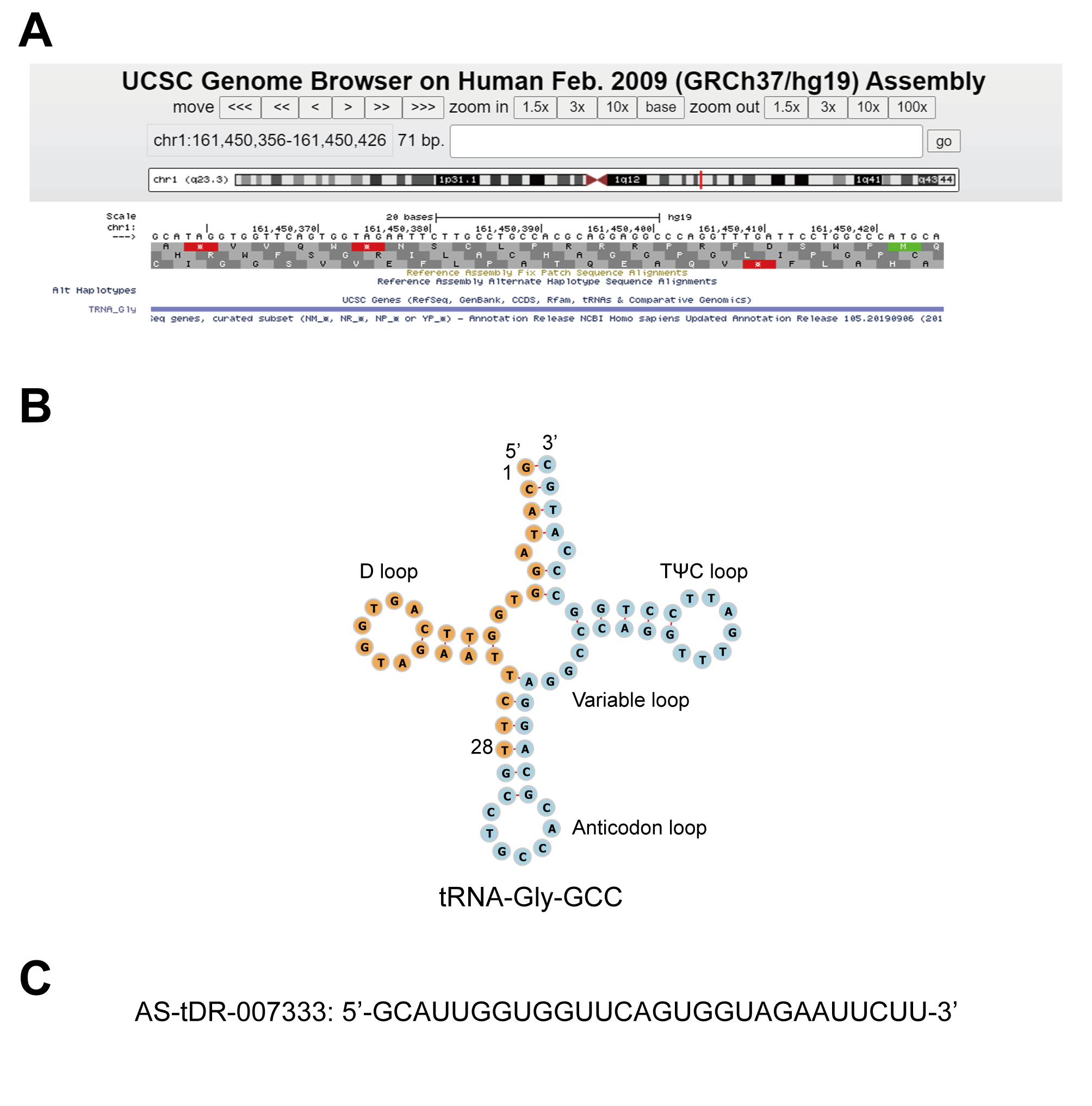

Supplement: Supplementary file 2 — Additional file 2: Figure S1. Characteristics of AS-tDR-007333. Figure S2. The transfection efficiencies of mimics, plasmids, and si-RNAs in NSCLC cells. Figure S3. AS-tDR-007333 did not affect apoptosis phenotypes in NSCLC cells. Figure S4. Gene set enrichment analysis (GSEA) of AS-tDR-007333-treated cells. Figure S5. In silico analysis of MED29 in NSCLC based on TCGA database. Figure S6. AS-tDR-007333 regulates MED29 expression and functionally interacts with MED29 in NSCLC cells. Figure S7. HSPB1 is up-regulated in NSCLC (in silico analysis based on TCGA database). Figure S8. CHX-chase assay results suggested that AS-tDR-007333 may not affect HSPB1 protein degradation. Figure S9. ELK4 was up-regulated in NSCLC based on TCGA database. Figure S10. ELK4 was up-regulated in NSCLC cells. Figure S11. Schematic diagram of genomic organization and chromatin state of the human MED29 gene locus. Figure S12. Overview of AS-tDR-007333 staining in tissue microarrays (TMAs) spots. Figure S13. AS-tDR-007333 inhibitor did not affect the body weight different subgroups of rats during the period of experiments. Figure S14. Correlations between AS-tDR-007333 and HSPB1, ELK4, and MED29 in NSCLC tumor tissues. [file 13045_2022_1270_MOESM2_ESM.zip › 13045_2022_1270_MOESM2_ESM/Figure S1.tif]

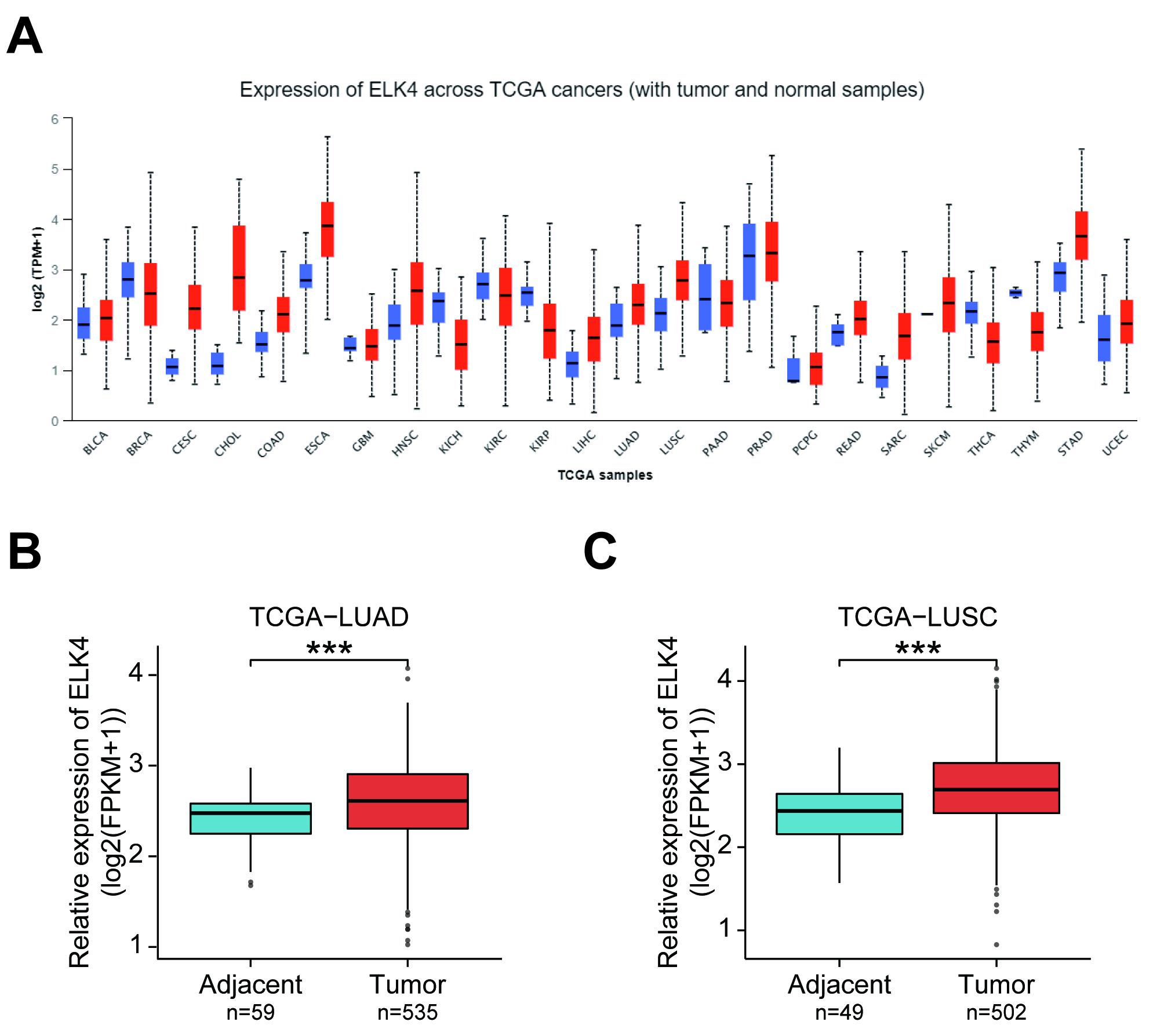

Supplement: Supplementary file 2 — Additional file 2: Figure S1. Characteristics of AS-tDR-007333. Figure S2. The transfection efficiencies of mimics, plasmids, and si-RNAs in NSCLC cells. Figure S3. AS-tDR-007333 did not affect apoptosis phenotypes in NSCLC cells. Figure S4. Gene set enrichment analysis (GSEA) of AS-tDR-007333-treated cells. Figure S5. In silico analysis of MED29 in NSCLC based on TCGA database. Figure S6. AS-tDR-007333 regulates MED29 expression and functionally interacts with MED29 in NSCLC cells. Figure S7. HSPB1 is up-regulated in NSCLC (in silico analysis based on TCGA database). Figure S8. CHX-chase assay results suggested that AS-tDR-007333 may not affect HSPB1 protein degradation. Figure S9. ELK4 was up-regulated in NSCLC based on TCGA database. Figure S10. ELK4 was up-regulated in NSCLC cells. Figure S11. Schematic diagram of genomic organization and chromatin state of the human MED29 gene locus. Figure S12. Overview of AS-tDR-007333 staining in tissue microarrays (TMAs) spots. Figure S13. AS-tDR-007333 inhibitor did not affect the body weight different subgroups of rats during the period of experiments. Figure S14. Correlations between AS-tDR-007333 and HSPB1, ELK4, and MED29 in NSCLC tumor tissues. [file 13045_2022_1270_MOESM2_ESM.zip › 13045_2022_1270_MOESM2_ESM/Figure S10.tif]

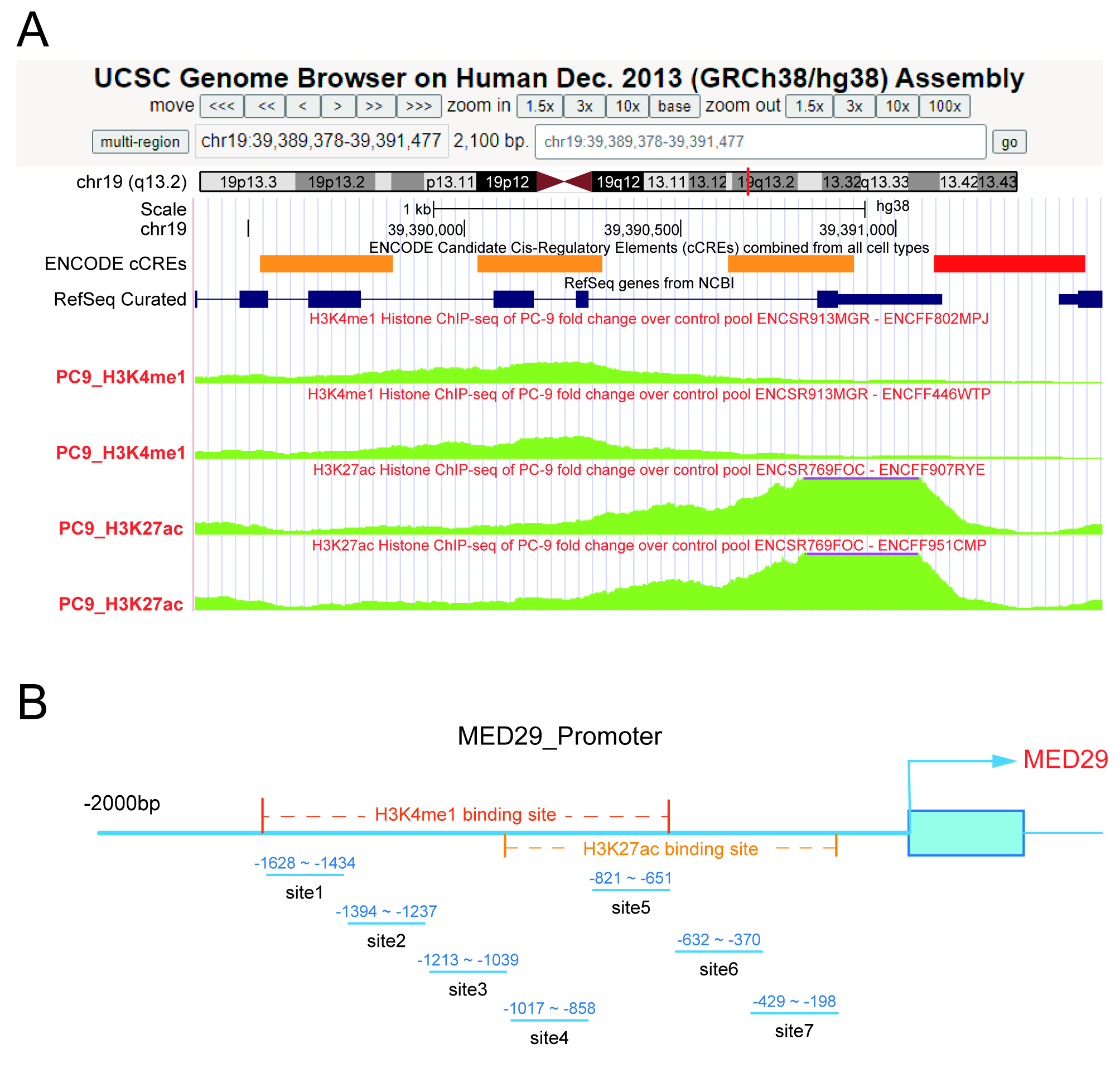

Supplement: Supplementary file 2 — Additional file 2: Figure S1. Characteristics of AS-tDR-007333. Figure S2. The transfection efficiencies of mimics, plasmids, and si-RNAs in NSCLC cells. Figure S3. AS-tDR-007333 did not affect apoptosis phenotypes in NSCLC cells. Figure S4. Gene set enrichment analysis (GSEA) of AS-tDR-007333-treated cells. Figure S5. In silico analysis of MED29 in NSCLC based on TCGA database. Figure S6. AS-tDR-007333 regulates MED29 expression and functionally interacts with MED29 in NSCLC cells. Figure S7. HSPB1 is up-regulated in NSCLC (in silico analysis based on TCGA database). Figure S8. CHX-chase assay results suggested that AS-tDR-007333 may not affect HSPB1 protein degradation. Figure S9. ELK4 was up-regulated in NSCLC based on TCGA database. Figure S10. ELK4 was up-regulated in NSCLC cells. Figure S11. Schematic diagram of genomic organization and chromatin state of the human MED29 gene locus. Figure S12. Overview of AS-tDR-007333 staining in tissue microarrays (TMAs) spots. Figure S13. AS-tDR-007333 inhibitor did not affect the body weight different subgroups of rats during the period of experiments. Figure S14. Correlations between AS-tDR-007333 and HSPB1, ELK4, and MED29 in NSCLC tumor tissues. [file 13045_2022_1270_MOESM2_ESM.zip › 13045_2022_1270_MOESM2_ESM/Figure S11.tif]

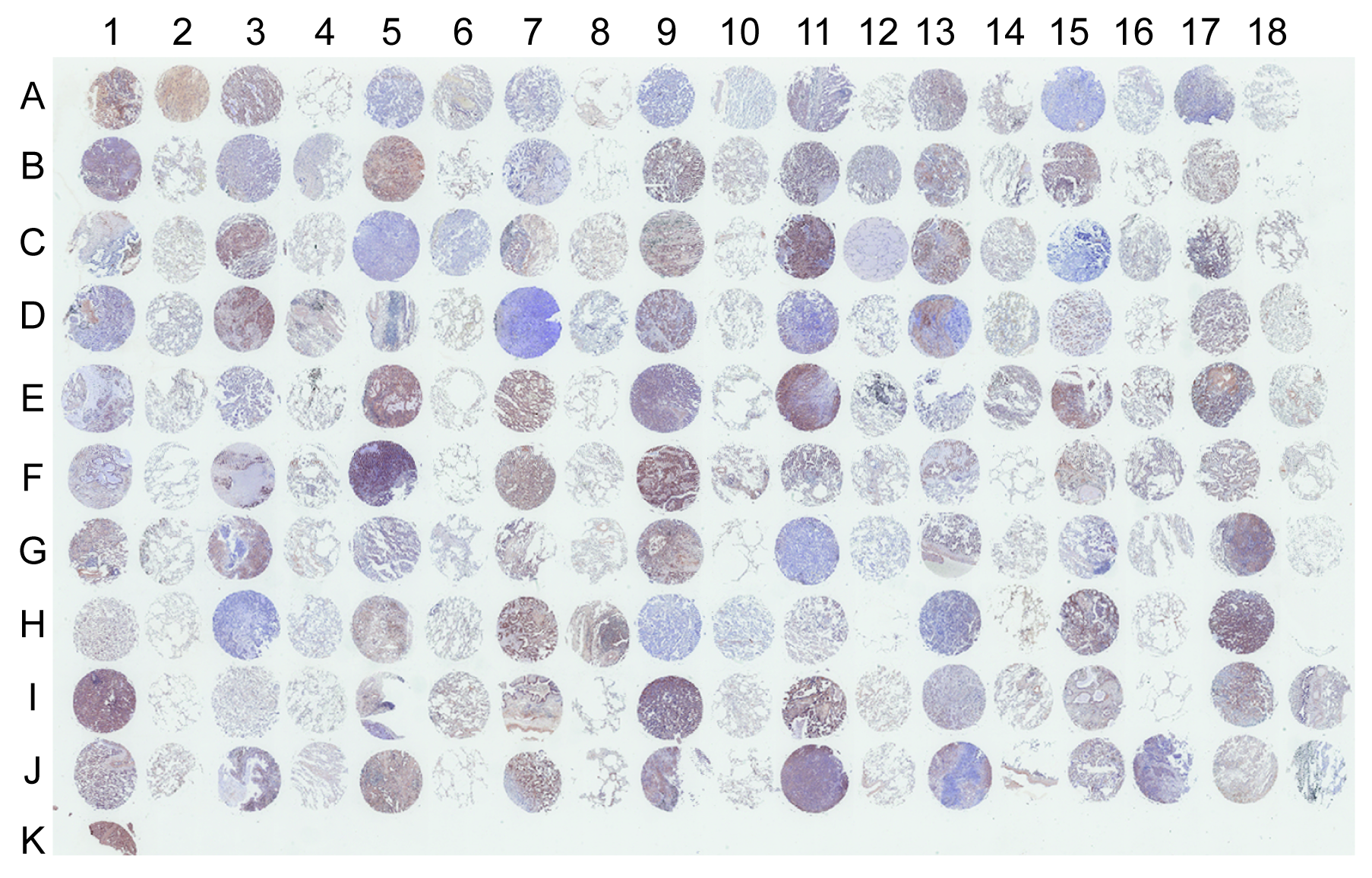

Supplement: Supplementary file 2 — Additional file 2: Figure S1. Characteristics of AS-tDR-007333. Figure S2. The transfection efficiencies of mimics, plasmids, and si-RNAs in NSCLC cells. Figure S3. AS-tDR-007333 did not affect apoptosis phenotypes in NSCLC cells. Figure S4. Gene set enrichment analysis (GSEA) of AS-tDR-007333-treated cells. Figure S5. In silico analysis of MED29 in NSCLC based on TCGA database. Figure S6. AS-tDR-007333 regulates MED29 expression and functionally interacts with MED29 in NSCLC cells. Figure S7. HSPB1 is up-regulated in NSCLC (in silico analysis based on TCGA database). Figure S8. CHX-chase assay results suggested that AS-tDR-007333 may not affect HSPB1 protein degradation. Figure S9. ELK4 was up-regulated in NSCLC based on TCGA database. Figure S10. ELK4 was up-regulated in NSCLC cells. Figure S11. Schematic diagram of genomic organization and chromatin state of the human MED29 gene locus. Figure S12. Overview of AS-tDR-007333 staining in tissue microarrays (TMAs) spots. Figure S13. AS-tDR-007333 inhibitor did not affect the body weight different subgroups of rats during the period of experiments. Figure S14. Correlations between AS-tDR-007333 and HSPB1, ELK4, and MED29 in NSCLC tumor tissues. [file 13045_2022_1270_MOESM2_ESM.zip › 13045_2022_1270_MOESM2_ESM/Figure S12.tif]

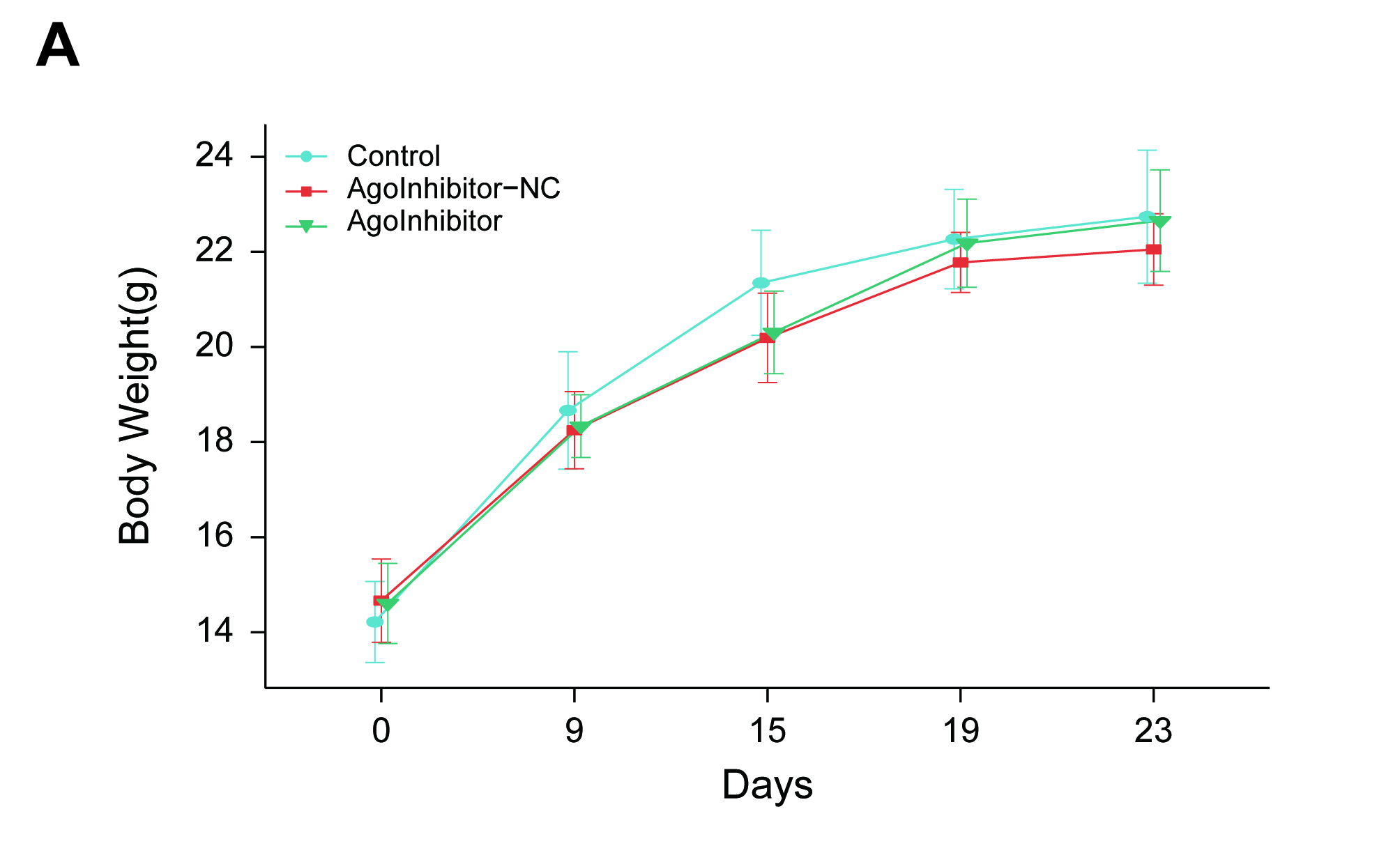

Supplement: Supplementary file 2 — Additional file 2: Figure S1. Characteristics of AS-tDR-007333. Figure S2. The transfection efficiencies of mimics, plasmids, and si-RNAs in NSCLC cells. Figure S3. AS-tDR-007333 did not affect apoptosis phenotypes in NSCLC cells. Figure S4. Gene set enrichment analysis (GSEA) of AS-tDR-007333-treated cells. Figure S5. In silico analysis of MED29 in NSCLC based on TCGA database. Figure S6. AS-tDR-007333 regulates MED29 expression and functionally interacts with MED29 in NSCLC cells. Figure S7. HSPB1 is up-regulated in NSCLC (in silico analysis based on TCGA database). Figure S8. CHX-chase assay results suggested that AS-tDR-007333 may not affect HSPB1 protein degradation. Figure S9. ELK4 was up-regulated in NSCLC based on TCGA database. Figure S10. ELK4 was up-regulated in NSCLC cells. Figure S11. Schematic diagram of genomic organization and chromatin state of the human MED29 gene locus. Figure S12. Overview of AS-tDR-007333 staining in tissue microarrays (TMAs) spots. Figure S13. AS-tDR-007333 inhibitor did not affect the body weight different subgroups of rats during the period of experiments. Figure S14. Correlations between AS-tDR-007333 and HSPB1, ELK4, and MED29 in NSCLC tumor tissues. [file 13045_2022_1270_MOESM2_ESM.zip › 13045_2022_1270_MOESM2_ESM/Figure S13.tif]

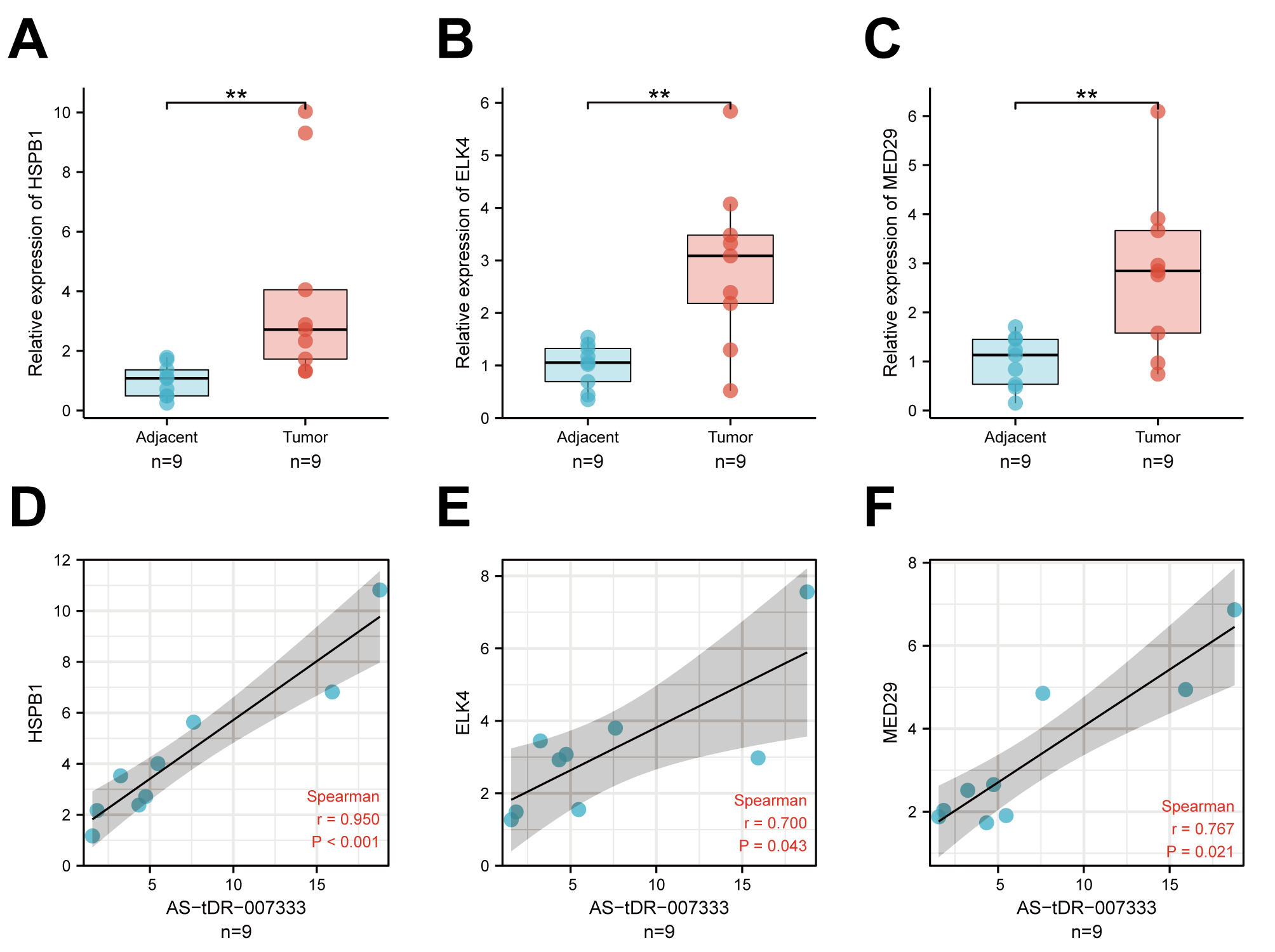

Supplement: Supplementary file 2 — Additional file 2: Figure S1. Characteristics of AS-tDR-007333. Figure S2. The transfection efficiencies of mimics, plasmids, and si-RNAs in NSCLC cells. Figure S3. AS-tDR-007333 did not affect apoptosis phenotypes in NSCLC cells. Figure S4. Gene set enrichment analysis (GSEA) of AS-tDR-007333-treated cells. Figure S5. In silico analysis of MED29 in NSCLC based on TCGA database. Figure S6. AS-tDR-007333 regulates MED29 expression and functionally interacts with MED29 in NSCLC cells. Figure S7. HSPB1 is up-regulated in NSCLC (in silico analysis based on TCGA database). Figure S8. CHX-chase assay results suggested that AS-tDR-007333 may not affect HSPB1 protein degradation. Figure S9. ELK4 was up-regulated in NSCLC based on TCGA database. Figure S10. ELK4 was up-regulated in NSCLC cells. Figure S11. Schematic diagram of genomic organization and chromatin state of the human MED29 gene locus. Figure S12. Overview of AS-tDR-007333 staining in tissue microarrays (TMAs) spots. Figure S13. AS-tDR-007333 inhibitor did not affect the body weight different subgroups of rats during the period of experiments. Figure S14. Correlations between AS-tDR-007333 and HSPB1, ELK4, and MED29 in NSCLC tumor tissues. [file 13045_2022_1270_MOESM2_ESM.zip › 13045_2022_1270_MOESM2_ESM/Figure S14.tif]

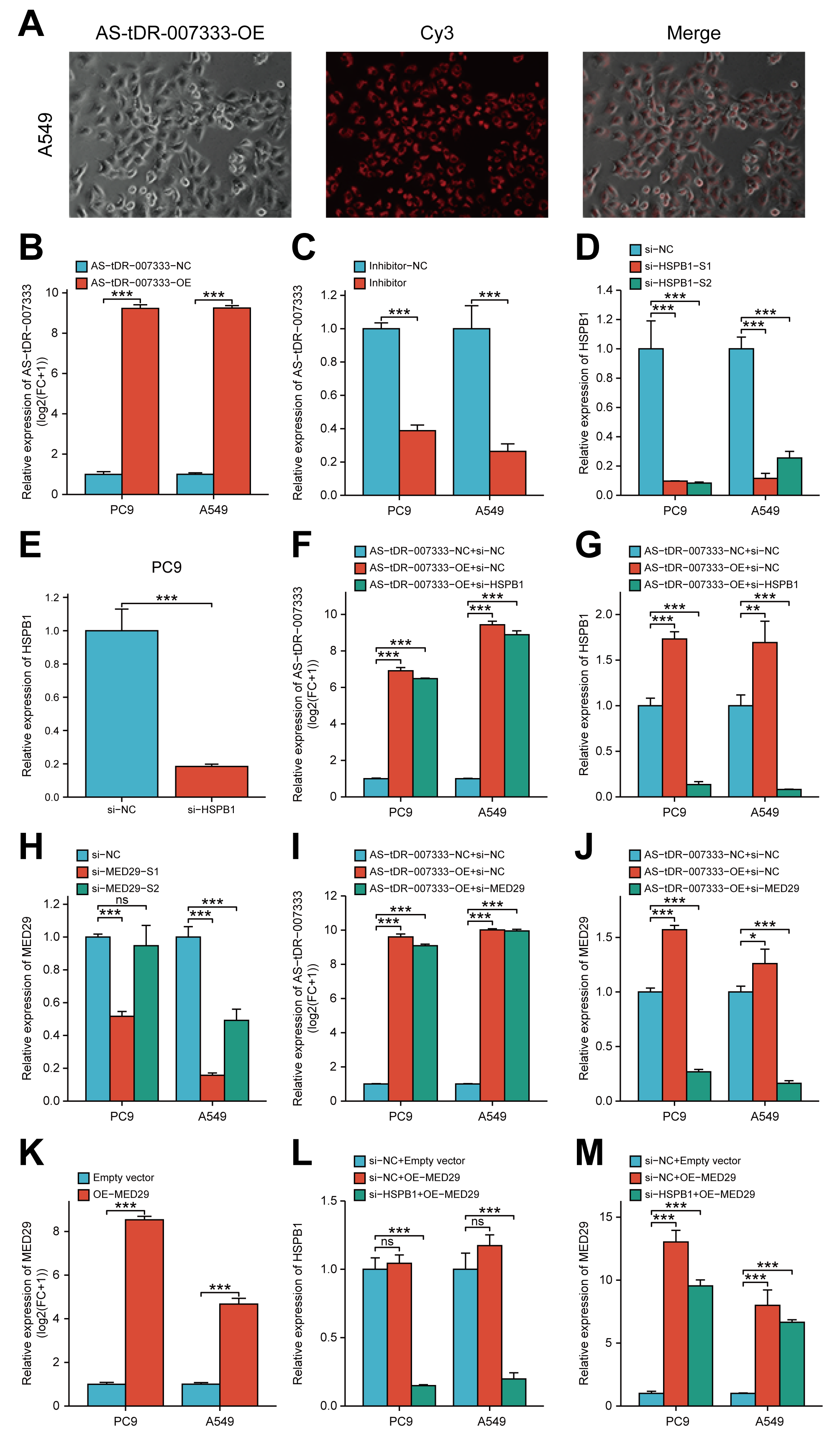

Supplement: Supplementary file 2 — Additional file 2: Figure S1. Characteristics of AS-tDR-007333. Figure S2. The transfection efficiencies of mimics, plasmids, and si-RNAs in NSCLC cells. Figure S3. AS-tDR-007333 did not affect apoptosis phenotypes in NSCLC cells. Figure S4. Gene set enrichment analysis (GSEA) of AS-tDR-007333-treated cells. Figure S5. In silico analysis of MED29 in NSCLC based on TCGA database. Figure S6. AS-tDR-007333 regulates MED29 expression and functionally interacts with MED29 in NSCLC cells. Figure S7. HSPB1 is up-regulated in NSCLC (in silico analysis based on TCGA database). Figure S8. CHX-chase assay results suggested that AS-tDR-007333 may not affect HSPB1 protein degradation. Figure S9. ELK4 was up-regulated in NSCLC based on TCGA database. Figure S10. ELK4 was up-regulated in NSCLC cells. Figure S11. Schematic diagram of genomic organization and chromatin state of the human MED29 gene locus. Figure S12. Overview of AS-tDR-007333 staining in tissue microarrays (TMAs) spots. Figure S13. AS-tDR-007333 inhibitor did not affect the body weight different subgroups of rats during the period of experiments. Figure S14. Correlations between AS-tDR-007333 and HSPB1, ELK4, and MED29 in NSCLC tumor tissues. [file 13045_2022_1270_MOESM2_ESM.zip › 13045_2022_1270_MOESM2_ESM/Figure S2.tif]

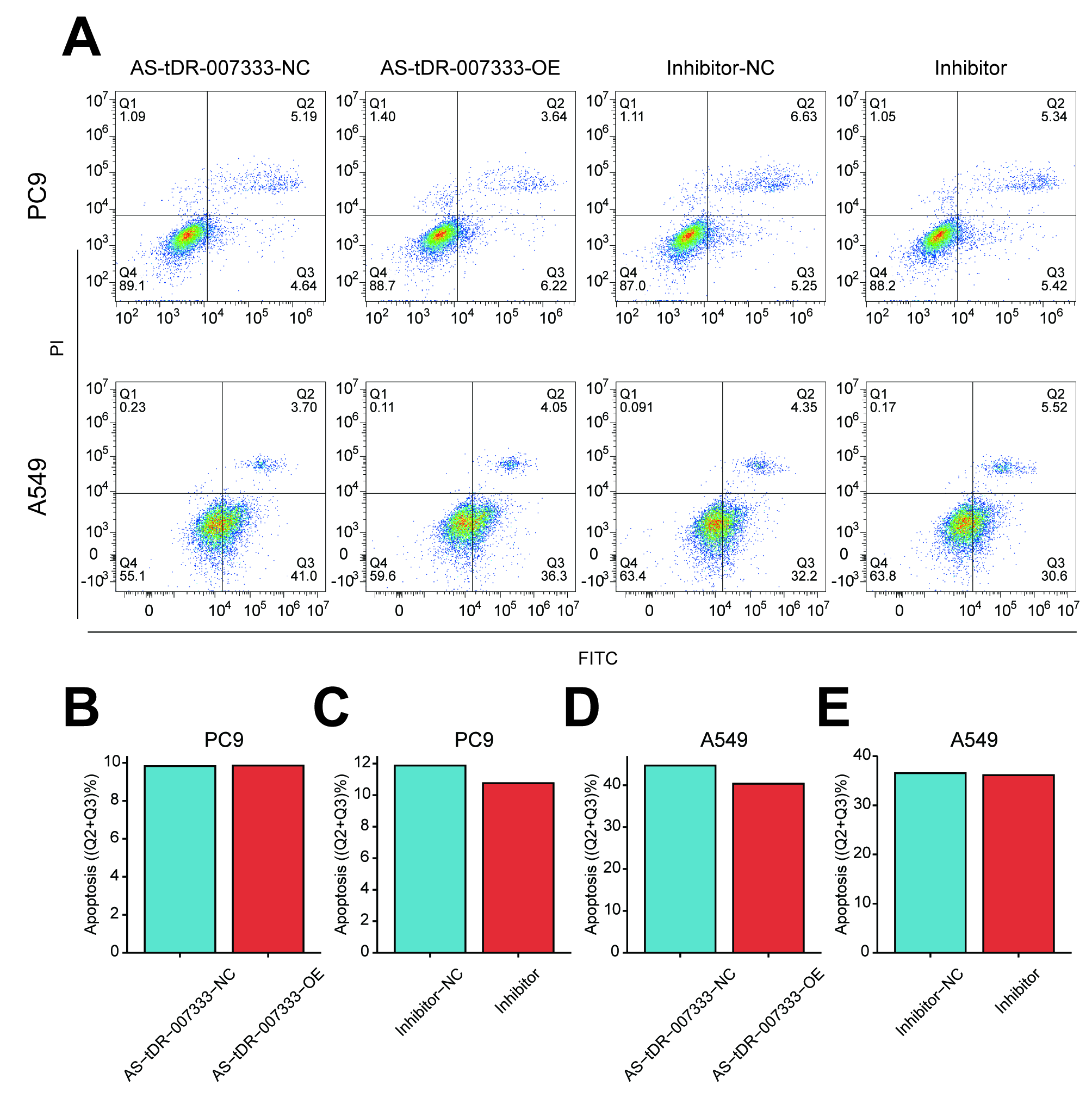

Supplement: Supplementary file 2 — Additional file 2: Figure S1. Characteristics of AS-tDR-007333. Figure S2. The transfection efficiencies of mimics, plasmids, and si-RNAs in NSCLC cells. Figure S3. AS-tDR-007333 did not affect apoptosis phenotypes in NSCLC cells. Figure S4. Gene set enrichment analysis (GSEA) of AS-tDR-007333-treated cells. Figure S5. In silico analysis of MED29 in NSCLC based on TCGA database. Figure S6. AS-tDR-007333 regulates MED29 expression and functionally interacts with MED29 in NSCLC cells. Figure S7. HSPB1 is up-regulated in NSCLC (in silico analysis based on TCGA database). Figure S8. CHX-chase assay results suggested that AS-tDR-007333 may not affect HSPB1 protein degradation. Figure S9. ELK4 was up-regulated in NSCLC based on TCGA database. Figure S10. ELK4 was up-regulated in NSCLC cells. Figure S11. Schematic diagram of genomic organization and chromatin state of the human MED29 gene locus. Figure S12. Overview of AS-tDR-007333 staining in tissue microarrays (TMAs) spots. Figure S13. AS-tDR-007333 inhibitor did not affect the body weight different subgroups of rats during the period of experiments. Figure S14. Correlations between AS-tDR-007333 and HSPB1, ELK4, and MED29 in NSCLC tumor tissues. [file 13045_2022_1270_MOESM2_ESM.zip › 13045_2022_1270_MOESM2_ESM/Figure S3.tif]

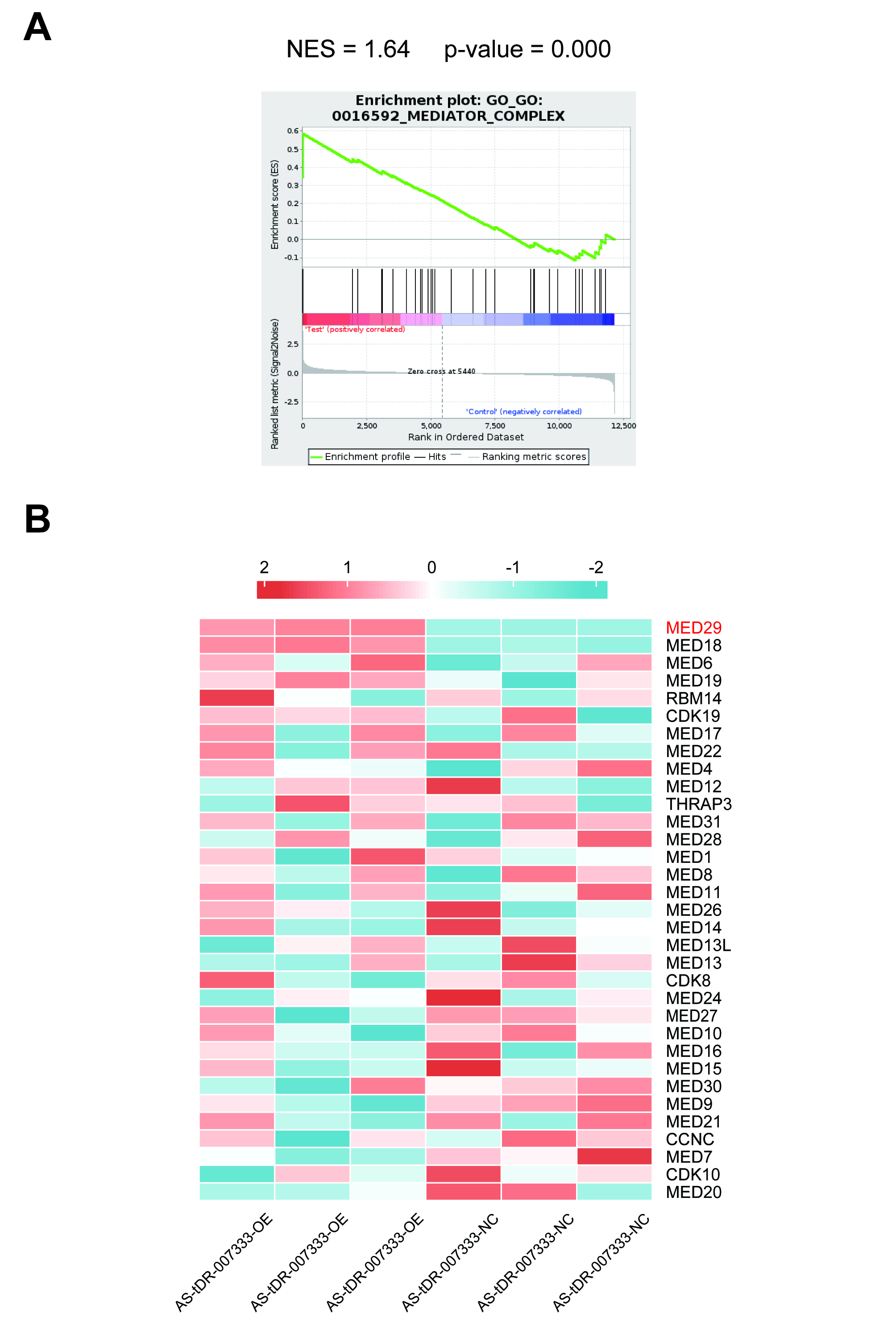

Supplement: Supplementary file 2 — Additional file 2: Figure S1. Characteristics of AS-tDR-007333. Figure S2. The transfection efficiencies of mimics, plasmids, and si-RNAs in NSCLC cells. Figure S3. AS-tDR-007333 did not affect apoptosis phenotypes in NSCLC cells. Figure S4. Gene set enrichment analysis (GSEA) of AS-tDR-007333-treated cells. Figure S5. In silico analysis of MED29 in NSCLC based on TCGA database. Figure S6. AS-tDR-007333 regulates MED29 expression and functionally interacts with MED29 in NSCLC cells. Figure S7. HSPB1 is up-regulated in NSCLC (in silico analysis based on TCGA database). Figure S8. CHX-chase assay results suggested that AS-tDR-007333 may not affect HSPB1 protein degradation. Figure S9. ELK4 was up-regulated in NSCLC based on TCGA database. Figure S10. ELK4 was up-regulated in NSCLC cells. Figure S11. Schematic diagram of genomic organization and chromatin state of the human MED29 gene locus. Figure S12. Overview of AS-tDR-007333 staining in tissue microarrays (TMAs) spots. Figure S13. AS-tDR-007333 inhibitor did not affect the body weight different subgroups of rats during the period of experiments. Figure S14. Correlations between AS-tDR-007333 and HSPB1, ELK4, and MED29 in NSCLC tumor tissues. [file 13045_2022_1270_MOESM2_ESM.zip › 13045_2022_1270_MOESM2_ESM/Figure S4.tif]

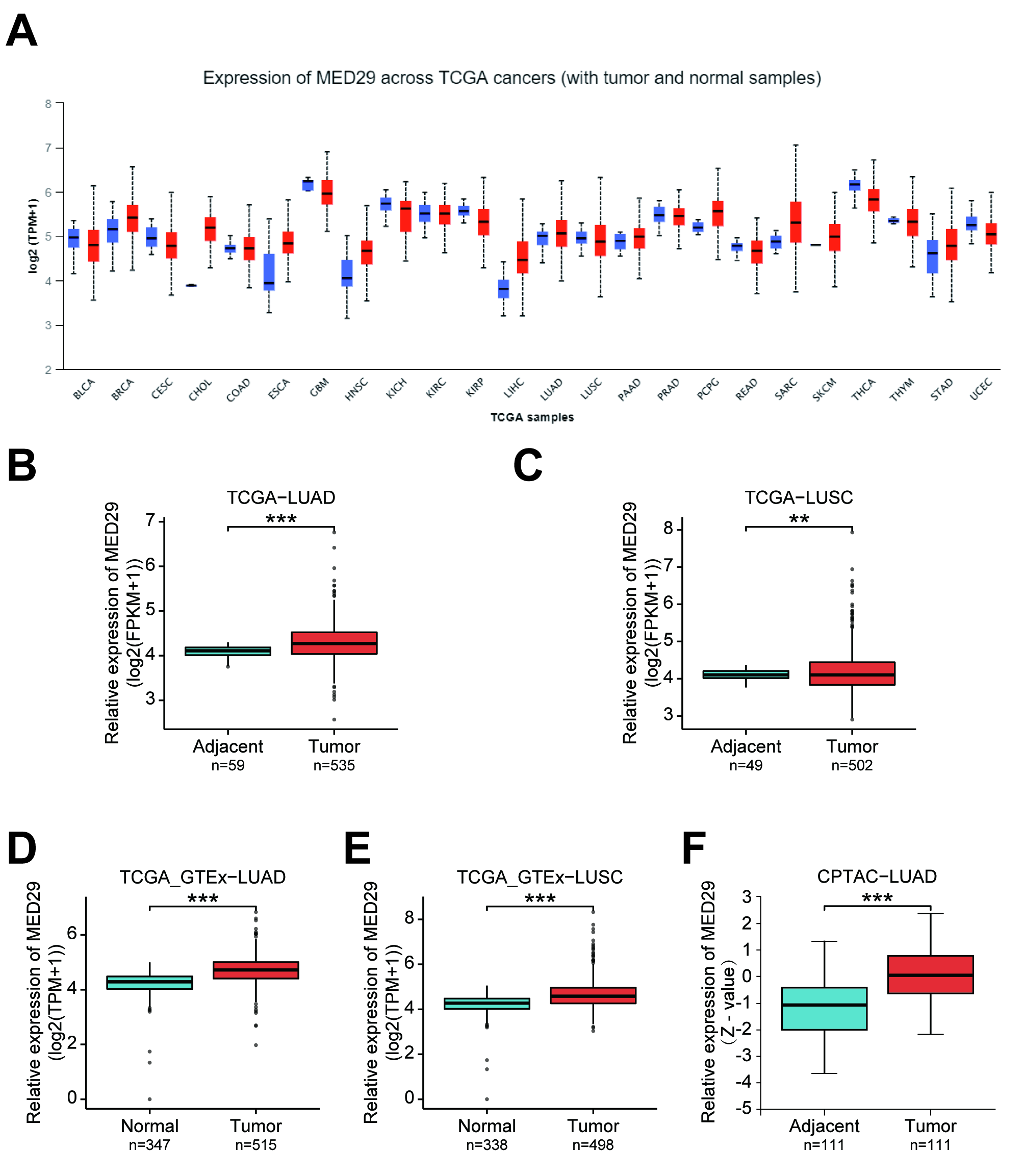

Supplement: Supplementary file 2 — Additional file 2: Figure S1. Characteristics of AS-tDR-007333. Figure S2. The transfection efficiencies of mimics, plasmids, and si-RNAs in NSCLC cells. Figure S3. AS-tDR-007333 did not affect apoptosis phenotypes in NSCLC cells. Figure S4. Gene set enrichment analysis (GSEA) of AS-tDR-007333-treated cells. Figure S5. In silico analysis of MED29 in NSCLC based on TCGA database. Figure S6. AS-tDR-007333 regulates MED29 expression and functionally interacts with MED29 in NSCLC cells. Figure S7. HSPB1 is up-regulated in NSCLC (in silico analysis based on TCGA database). Figure S8. CHX-chase assay results suggested that AS-tDR-007333 may not affect HSPB1 protein degradation. Figure S9. ELK4 was up-regulated in NSCLC based on TCGA database. Figure S10. ELK4 was up-regulated in NSCLC cells. Figure S11. Schematic diagram of genomic organization and chromatin state of the human MED29 gene locus. Figure S12. Overview of AS-tDR-007333 staining in tissue microarrays (TMAs) spots. Figure S13. AS-tDR-007333 inhibitor did not affect the body weight different subgroups of rats during the period of experiments. Figure S14. Correlations between AS-tDR-007333 and HSPB1, ELK4, and MED29 in NSCLC tumor tissues. [file 13045_2022_1270_MOESM2_ESM.zip › 13045_2022_1270_MOESM2_ESM/Figure S5.tif]

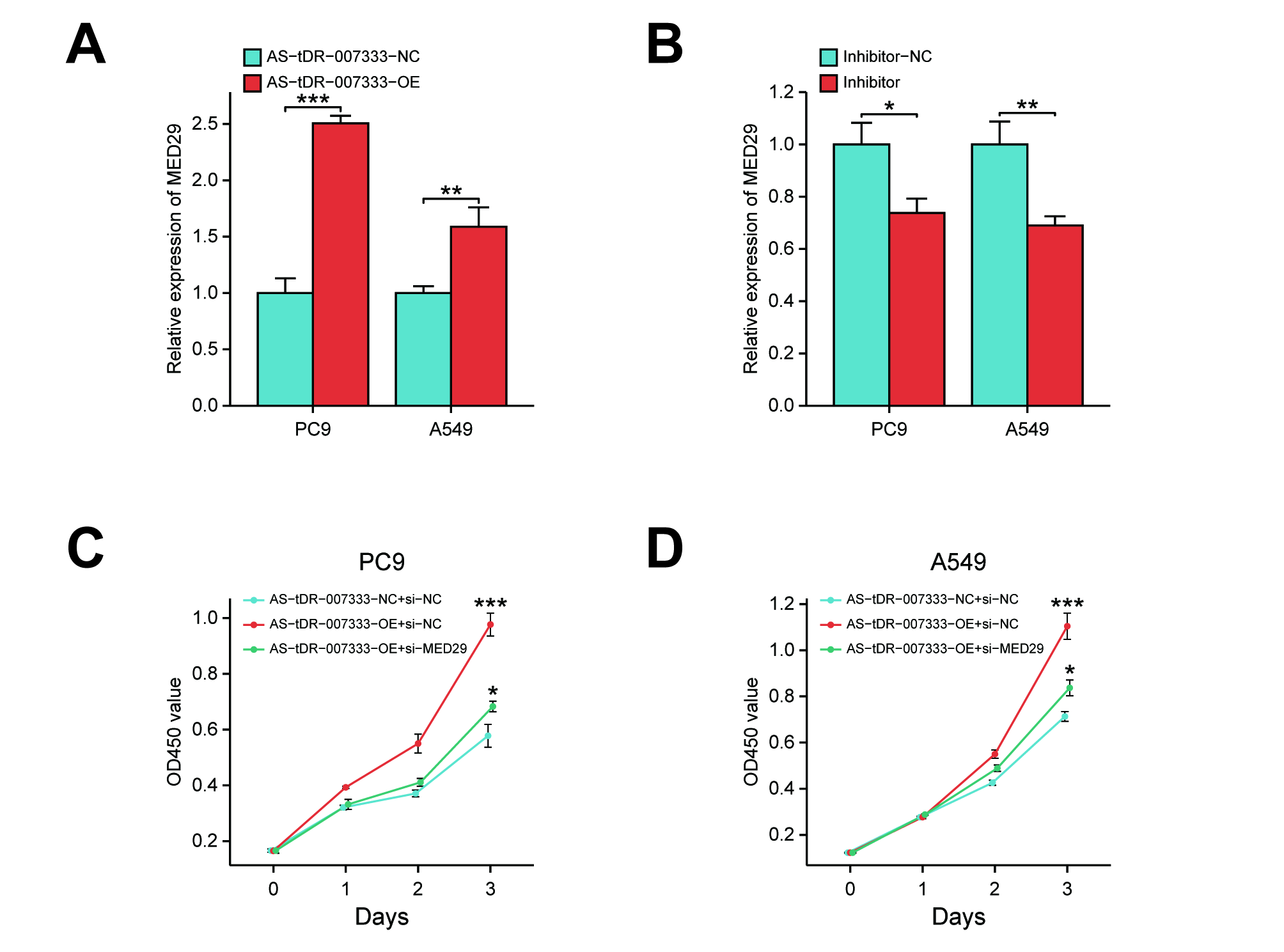

Supplement: Supplementary file 2 — Additional file 2: Figure S1. Characteristics of AS-tDR-007333. Figure S2. The transfection efficiencies of mimics, plasmids, and si-RNAs in NSCLC cells. Figure S3. AS-tDR-007333 did not affect apoptosis phenotypes in NSCLC cells. Figure S4. Gene set enrichment analysis (GSEA) of AS-tDR-007333-treated cells. Figure S5. In silico analysis of MED29 in NSCLC based on TCGA database. Figure S6. AS-tDR-007333 regulates MED29 expression and functionally interacts with MED29 in NSCLC cells. Figure S7. HSPB1 is up-regulated in NSCLC (in silico analysis based on TCGA database). Figure S8. CHX-chase assay results suggested that AS-tDR-007333 may not affect HSPB1 protein degradation. Figure S9. ELK4 was up-regulated in NSCLC based on TCGA database. Figure S10. ELK4 was up-regulated in NSCLC cells. Figure S11. Schematic diagram of genomic organization and chromatin state of the human MED29 gene locus. Figure S12. Overview of AS-tDR-007333 staining in tissue microarrays (TMAs) spots. Figure S13. AS-tDR-007333 inhibitor did not affect the body weight different subgroups of rats during the period of experiments. Figure S14. Correlations between AS-tDR-007333 and HSPB1, ELK4, and MED29 in NSCLC tumor tissues. [file 13045_2022_1270_MOESM2_ESM.zip › 13045_2022_1270_MOESM2_ESM/Figure S6.tif]

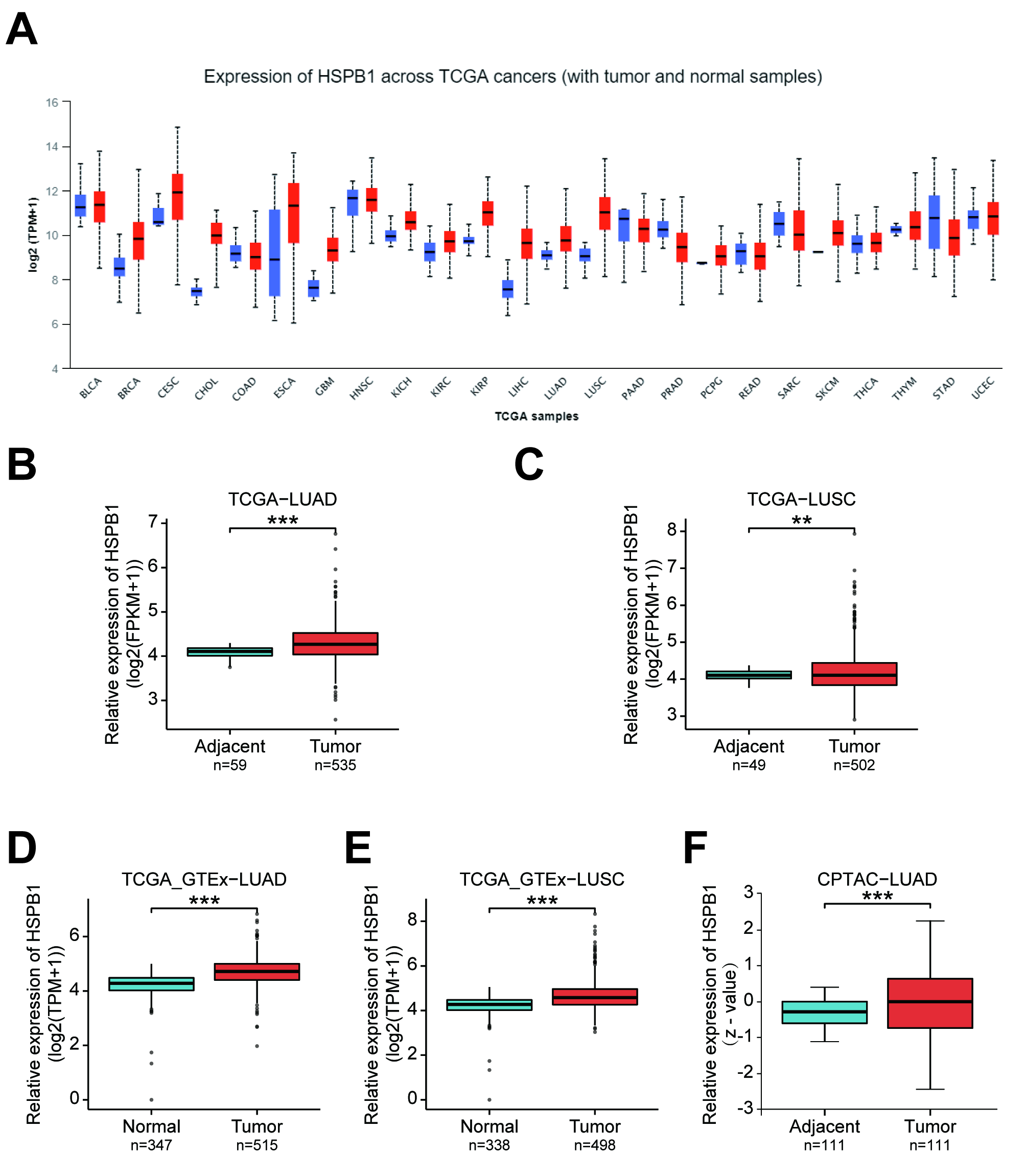

Supplement: Supplementary file 2 — Additional file 2: Figure S1. Characteristics of AS-tDR-007333. Figure S2. The transfection efficiencies of mimics, plasmids, and si-RNAs in NSCLC cells. Figure S3. AS-tDR-007333 did not affect apoptosis phenotypes in NSCLC cells. Figure S4. Gene set enrichment analysis (GSEA) of AS-tDR-007333-treated cells. Figure S5. In silico analysis of MED29 in NSCLC based on TCGA database. Figure S6. AS-tDR-007333 regulates MED29 expression and functionally interacts with MED29 in NSCLC cells. Figure S7. HSPB1 is up-regulated in NSCLC (in silico analysis based on TCGA database). Figure S8. CHX-chase assay results suggested that AS-tDR-007333 may not affect HSPB1 protein degradation. Figure S9. ELK4 was up-regulated in NSCLC based on TCGA database. Figure S10. ELK4 was up-regulated in NSCLC cells. Figure S11. Schematic diagram of genomic organization and chromatin state of the human MED29 gene locus. Figure S12. Overview of AS-tDR-007333 staining in tissue microarrays (TMAs) spots. Figure S13. AS-tDR-007333 inhibitor did not affect the body weight different subgroups of rats during the period of experiments. Figure S14. Correlations between AS-tDR-007333 and HSPB1, ELK4, and MED29 in NSCLC tumor tissues. [file 13045_2022_1270_MOESM2_ESM.zip › 13045_2022_1270_MOESM2_ESM/Figure S7.tif]

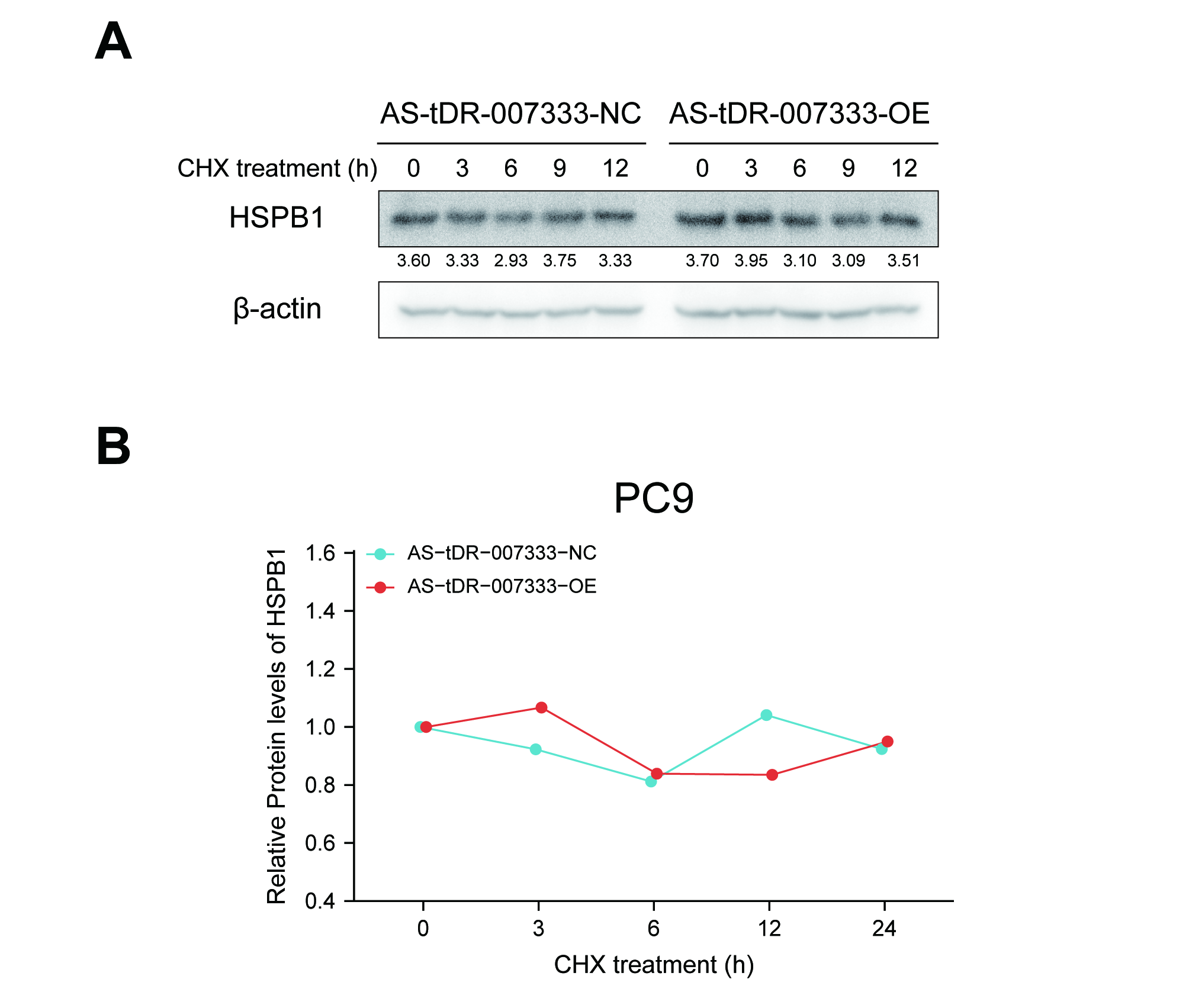

Supplement: Supplementary file 2 — Additional file 2: Figure S1. Characteristics of AS-tDR-007333. Figure S2. The transfection efficiencies of mimics, plasmids, and si-RNAs in NSCLC cells. Figure S3. AS-tDR-007333 did not affect apoptosis phenotypes in NSCLC cells. Figure S4. Gene set enrichment analysis (GSEA) of AS-tDR-007333-treated cells. Figure S5. In silico analysis of MED29 in NSCLC based on TCGA database. Figure S6. AS-tDR-007333 regulates MED29 expression and functionally interacts with MED29 in NSCLC cells. Figure S7. HSPB1 is up-regulated in NSCLC (in silico analysis based on TCGA database). Figure S8. CHX-chase assay results suggested that AS-tDR-007333 may not affect HSPB1 protein degradation. Figure S9. ELK4 was up-regulated in NSCLC based on TCGA database. Figure S10. ELK4 was up-regulated in NSCLC cells. Figure S11. Schematic diagram of genomic organization and chromatin state of the human MED29 gene locus. Figure S12. Overview of AS-tDR-007333 staining in tissue microarrays (TMAs) spots. Figure S13. AS-tDR-007333 inhibitor did not affect the body weight different subgroups of rats during the period of experiments. Figure S14. Correlations between AS-tDR-007333 and HSPB1, ELK4, and MED29 in NSCLC tumor tissues. [file 13045_2022_1270_MOESM2_ESM.zip › 13045_2022_1270_MOESM2_ESM/Figure S8.tif]
